# An anti-PD-1–GITR-L bispecific agonist induces GITR clustering-mediated T cell activation for cancer immunotherapy

**DOI:** 10.1038/s43018-022-00334-9

**Published:** 2022-03-07

**Authors:** Sarah Chan, Nicole Belmar, Sun Ho, Bryan Rogers, Marcia Stickler, Michelle Graham, Eileen Lee, Ninian Tran, Dong Zhang, Priyanka Gupta, Mien Sho, Tracy MacDonough, Andrew Woolley, Han Kim, Hong Zhang, Wei Liu, Pingping Zheng, Zoltan Dezso, Kyle Halliwill, Michele Ceccarelli, Susan Rhodes, Archana Thakur, Charles M. Forsyth, Mengli Xiong, Siu Sze Tan, Ramesh Iyer, Marc Lake, Enrico Digiammarino, Li Zhou, Lance Bigelow, Kenton Longenecker, Russell A. Judge, Cassie Liu, Max Trumble, Jonathan P. Remis, Melvin Fox, Belinda Cairns, Yoshiko Akamatsu, Diane Hollenbaugh, Fiona Harding, Hamsell M. Alvarez

**Affiliations:** 1 AbbVie Redwood City, Redwood City, CA USA; 2 Calico Labs, South San Francisco, CA USA; 3 Seattle Genetics, South San Francisco, CA USA; 4 Atomwise, San Francisco, CA USA; 5 Bristol Myers Squibb, Redwood City, CA USA; 6 Bolt Biotherapeutics, Inc., Redwood City, CA USA; 7 Sanofi, Cambridge, MA USA; 8 University of Naples Federico II, Naples, Italy; 9 AbbVie, Inc., North Chicago, IL USA; 10 AbbVie Bioresearch Center, Worcester, MA USA; 11 Former Department of Molecular Biosciences, Northwestern University, Evanston, IL USA; 12 California Institute for Quantitative Biosciences, University of California, Berkeley, CA USA; 13 Oxford Biotherapeutics, San Jose, CA USA; 14 Good Therapeutics, Inc., Seattle, WA USA

**Keywords:** Cancer, Cancer immunotherapy, Drug development, Antibody therapy

## Abstract

Costimulatory receptors such as glucocorticoid-induced tumor necrosis factor receptor–related protein (GITR) play key roles in regulating the effector functions of T cells. In human clinical trials, however, GITR agonist antibodies have shown limited therapeutic effect, which may be due to suboptimal receptor clustering-mediated signaling. To overcome this potential limitation, a rational protein engineering approach is needed to optimize GITR agonist-based immunotherapies. Here we show a bispecific molecule consisting of an anti-PD-1 antibody fused with a multimeric GITR ligand (GITR-L) that induces PD-1-dependent and FcγR-independent GITR clustering, resulting in enhanced activation, proliferation and memory differentiation of primed antigen-specific GITR^+^PD-1^+^ T cells. The anti-PD-1–GITR-L bispecific is a PD-1-directed GITR-L construct that demonstrated dose-dependent, immunologically driven tumor growth inhibition in syngeneic, genetically engineered and xenograft humanized mouse tumor models, with a dose-dependent correlation between target saturation and Ki67 and TIGIT upregulation on memory T cells. Anti-PD-1–GITR-L thus represents a bispecific approach to directing GITR agonism for cancer immunotherapy.

## Abstract (web summary)

Alvarez and colleagues develop a bispecific anti-PD-1–GITR-L agonist that activates T cells via a mechanism distinct from those found with individual PD-1 and GITR-L agonists and demonstrate its antitumor activity in mice and nonhuman primates.

## Main

PD-1 is an inhibitory receptor expressed on T cells during the priming and effector phases of the adaptive immune response. PD-1 downregulates T cell activity upon binding to PD-L1 or PD-L2 by induction of a dominant negative checkpoint signal that limits subsequent antigen receptor-driven cellular activation. PD-1 signals through the phosphorylation of ITSM and recruitment of the tyrosine phosphatase SHP-2, which leads to inhibition of PI3K and Akt signaling^1^. Antibody-mediated inhibition of the PD-1 checkpoint signal results in prolonged antigen-specific T cell activation in vitro and an enhanced antitumor response in mouse models^2^. Although the clinical activity of anti-PD-1 therapy has shown durable antitumor immune responses in multiple indications^3^, not all patients treated with PD-1-targeted therapy experience tumor shrinkage, durable response or prolonged survival^4^.

GITR is a costimulatory member of the tumor necrosis factor receptor superfamily (TNFRSF) that plays a critical role in the enhancement of nascent immune responses. GITR is upregulated on activated T cells, constitutively expressed on regulatory T cells (T_regs_) and expressed at low levels on natural killer (NK) cells. Ligand binding to GITR modulates the NFκB and MAPK pathways. GITR signaling results in T cell activation, proliferation, survival and inhibition of the suppressive activity of T_regs_^5^. Multiple studies in mice have demonstrated that GITR ligation by either an agonistic antibody or a GITR-L construct induces an immunological response and increases resistance to tumors by accumulation of CD8^+^ T cells and intratumoral T_reg_ cell depletion^6–8^. In humans, even though anti-GITR antibodies have shown acceptable tolerability and reduction in circulating and intratumoral T_regs_ by induction of apoptosis or antibody-dependent cellular cytotoxicity (ADCC), their therapeutic effect has proved limited in several clinical trials^9–11^. In comparison, a minimally depleting anti-GITR isotype antibody has also shown limited bioactivity in humans, potentially due to suboptimal GITR crosslinking^12^. These results suggest that anti-GITR-mediated T_reg_ cell depletion is not sufficient to induce survival in humans and that the limited bioactivity is potentially due to a lack of T cell activation/proliferation, mediated by optimal GITR receptor clustering and other inhibitory signaling mechanisms^13^.

Since PD-1 and GITR are coexpressed on antigen-activated and memory T cells, and it has been shown that anti-PD-1 and anti-GITR antibodies induce crossregulation of PD-1 and GITR expression^14^, a bispecific construct that targets both specificities is warranted. The anti-PD-1–GITR-L will overcome immune escape to PD-(L)1 blockade by enhancing and sustaining GITR-L-mediated T cell activation, proliferation and memory differentiation of primed antigen-specific T cells by inducing PD-1 mediated GITR clustering in cis. The proposed mechanism of action of this bispecific is different from either the individual contributions of anti-PD-1 and anti-GITR monotherapies or their combination, because it is not reliant on PD-1 saturation, FcγR-mediated T cell activation or ADCC-mediated T_reg_ depletion. The anti-PD-1–GITR-L bispecific represents a different approach for T cell agonism in cancer immunotherapy.

## Results

### GITR clustering is critical to induction of T cell activation

**Fig. 1:**
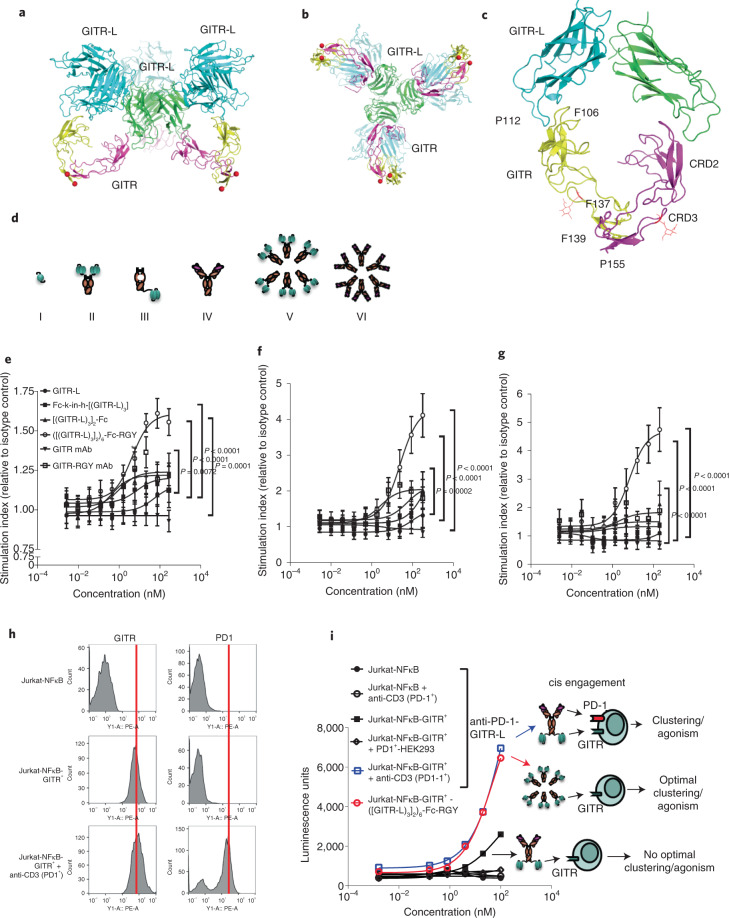
GITR clustering is crucial for induction of human T cell activation. **a**,**b**, Side (**a**) and top view (**b**) of the GITR–GITR-L complex structure solved at 2.75 Å. Structure centered around a trimeric GITR-L (green), with three GITR monomeric molecules (magenta) each noncovalently dimerized to a GITR (yellow) interacting with neighboring GITR-L trimers (light blue). N-linked glycosylation sites of GITR are shown as red spheres. **c**, The CRD3 domain of GITR mediates noncovalent dimerization through phenylalanine (F137 and F139) and proline (P155) residues. N-linked glycosylations are represented by red lines. **d**, Schematic representation of engineered constructs (percentage aggregation). I, Monomeric GITR-L (2%); II, bivalent [(GITR-L)_3_]_2_-Fc (1%); III, monovalent Fc-k-in-h-(GITR-L)_3_ (0%); IV, anti-GITR mAb (2%); V, dodecavalent Fc-GITR-L (3%); and VI, dodecavalent anti-GITR (2%). All constructs contain LALA, while hexamer constructs contain RGY in addition to LALA. **e**–**g**, Human PBMC costimulation assay (*n* = 5 donors) following indicated treatments in the presence of anti-CD3. Cells and supernatants were harvested/collected to assess cell viability (*t* = 96 h) (**e**), IL-2 (*t* = 48 h) (**f**) and IFN-γ (*t* = 96 h) (**g**). Data presented as mean ± s.e.m. Statistical significance was calculated by two-way analysis of variance (ANOVA) with Tukey’s correction for multiple comparisons (statistic refers to hexameric Fc-GITR-L versus monomeric GITR-L) (*n* = 2 technical cell culture replicates within a single experiment). **h**, GITR and PD-1 expression on transfected and activated Jurkat-NFκB-huGITR^+^ reporter cells (representative data of *n* = 2 independent experiments with similar results). **i**, NFκB signaling in an anti-CD3-activated Jurkat-NFκB-huGITR^+^ reporter assay following threefold titration with the anti-huPD-1-huGITR-L bispecific. A dodecavalent Fc-huGITR-L construct (with RGY and LALA mutations) was utilized as a positive control using Jurkat-NFκB-huGITR^+^ cells (indicated by red circles) (*n* = 2 technical cell culture replicates within a single experiment). Source data

**Extended Data Fig. 1:**
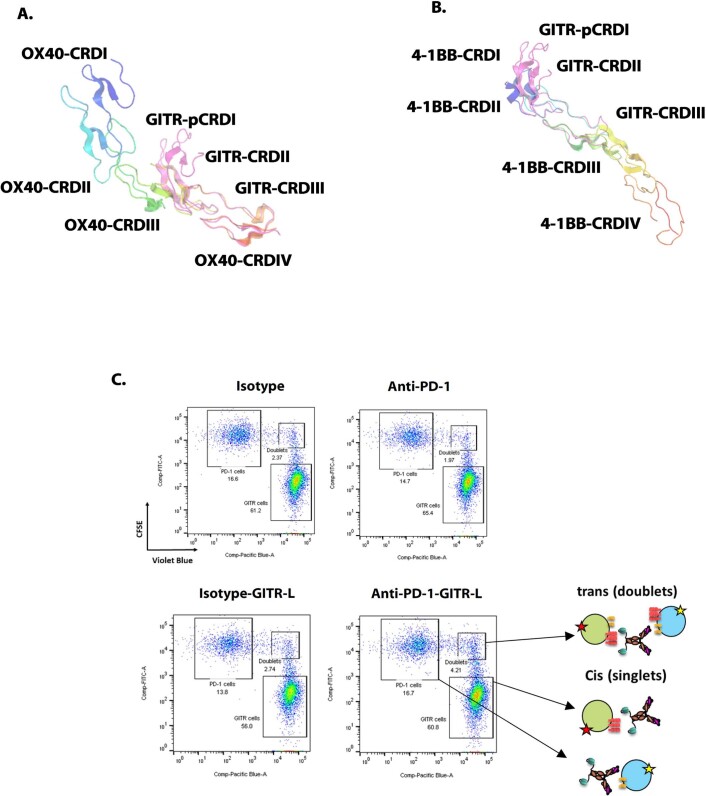
GITR structural alignment comparison with other TNFR members and flow cytometry cell bridging assay with the anti-PD-1-GITR-L bispecific. (**A** and **B**) GITR (magenta) overlaid with OX40 (PDB: 2HEV, rainbow, RMS 1.3) (**A**) and 4-1BB (PDB: 6BWV, rainbow, RMS 1.1) (**B**). Note that CRDI of GITR (pCRD1_GITR_) is only partially resolved due to compositional heterogeneity. (**C**) A 1:1 combination of CFSE-labeled PD-1-HEK293 and Violet-blue-labeled GITR-HEK293 cells were treated with 2.5 µgs/ml of isotype control, anti-PD-1 mAb, isotype-GITR-L construct and anti-PD-1-GITR-L bispecific for 30 mins.

While the crystal structure of GITR-L has been solved elsewhere^15,16^, we report here the human GITR-L–GITR (huGITR-L–GITR) complex at 2.75 Å. The structure reveals a dimer of GITR receptors where each receptor binds to a GITR-L monomer belonging to a separate GITR-L trimeric unit (Fig. 1a,b). Noncovalent receptor dimerization is observed at a hydrophobic interface mediated by several aromatic residues, including Phe137 and Phe139, from each CRD3 domain (Fig. 1c)^17^. The receptor–ligand interface is composed of GITR residues 103–109 with Phe106, that contacts a tip of the GITR-L surface centered at Asn53 and Pro112. The structure includes GITR residues 74–156 modeled with glycosylation on Asn146, while residues 61–73 are only partially resolved. The GITR subunit in the complex shows structural similarity to other members of the TNFRSF (Extended Data Fig. 1a,b)^18,19^. Likewise, the crystalline lattice network is reminiscent of previously published structures of TNFR1 and 4-1BB^20,21^. Noncovalent GITR-L-mediated receptor homodimerization is probably capable of dynamics not captured here and may represent just one of multiple states on a path necessary for optimal GITR receptor clustering.

To evaluate the effect of T cell costimulation corresponding to the levels of receptor clustering, we engineered a series of constructs (Fig. 1d). All constructs contain an effector-null Fc (L234A and L235A (LALA)) domain. Either trimeric GITR-L or anti-GITR was fused to an Fc containing the E345R, E430G and S440Y (RGY) mutations previously reported to enhance hexamerization^22^. We demonstrated that optimal oligomerization and bioactivity can be achieved using these engineered GITR-L- and anti-GITR-based constructs. A significant increase in T cell viability and secretion of interleukin 2 (IL-2) and interferon (IFN)-γ was observed only with the dodecavalent GITR-L hexameric Fc construct in the presence of anti-CD3 (Fig. 1e–g). Soluble monomeric GITR-L and anti-GITR showed limited levels of T cell activation. Increased numbers of GITR-L in the construct resulted in a higher level of costimulation (Fc-knob-into-hole (Fc-k-in-h)-(GITR-L)_3_ and divalent Fc-[(GITR-L)_3_]_2_). Dodecavalent anti-GITR hexameric antibody showed higher activity than the anti-GITR antibody, albeit at significantly lower levels than the hexameric ligand–trimer fusion. These results indicate that GITR signaling can be induced independently of FcγR binding when efficient GITR clustering is achieved by the strong avidity provided by multiple ligands, as previously suggested for other members of the TNFRSF^23^. With this mechanism in consideration, we designed an anti-PD-1–GITR-L bispecific where anti-PD-1 enhances GITR clustering and NFκB signaling of primed, antigen-specific T cells in cis. The anti-huPD-1-huGITR-L induced a substantial increase in NFκB signaling at higher concentrations only in an anti-CD3-activated NFκB-GITR^+^ Jurkat reporter cell line (where PD-1 is upregulated) (Fig. 1h), due to anti-PD-1-dependent GITR clustering (Fig. 1i). Similar NFκB signaling has been observed with the dodecavalent Fc-GITR-L construct using resting Jurkat-NFκB-huGITR^+^ cells. This Jurkat reporter cell line is not sensitive to anti-PD-1 blockade due to absence of PD-L1 expression. Also, lack of activity has been shown following treatment of Jurkat NFkB cells with anti-CD3 and the anti-PD-1–GITR-L bispecific, which suggests that anti-CD3 does not induce GITR-mediated signaling. It has also been shown that the anti-PD-1–GITR-L bispecific preferentially mediates PD-1-mediated GITR clustering in cis more than in trans, based on the absence of notable trans engagement (doublets and signaling) observed in a bridging assay, and in a combination of Jurkat-NFκB-huGITR^+^ and huPD-1-HEK293 cells (Extended Data Fig. 1c,i).

### Coexpression of PD-1 and GITR on human T cells

**Fig. 2:**
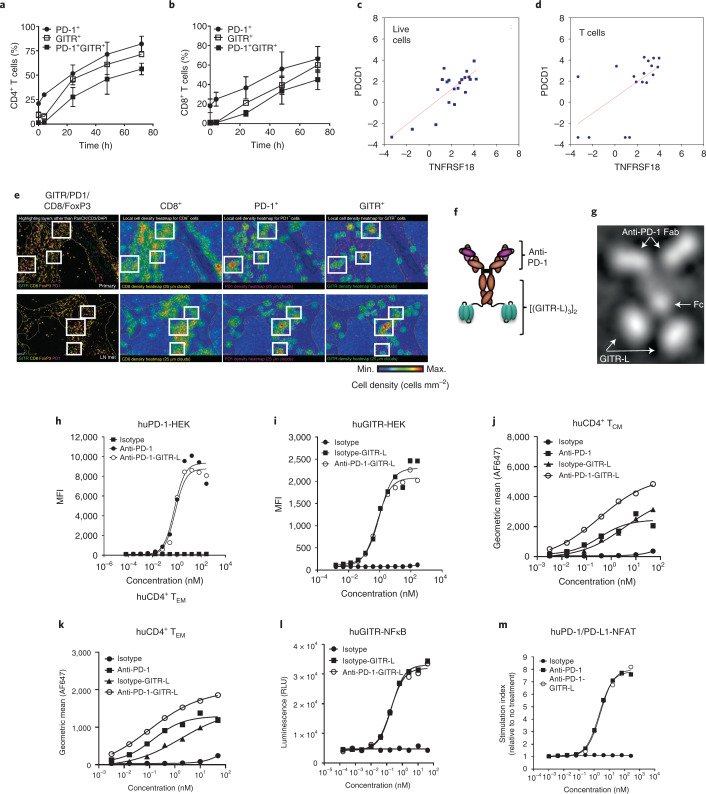
Expression of PD-1 and GITR in human T cells and in vitro characterization of anti-huPD-1-huGITR-L bispecific fusion protein. **a**–**d**, Percentage of PD-1 and GITR double-positive T cells following anti-CD3/CD28 activation of human CD4^+^ (**a**) and CD8^+^ T cells (**b**) (*n* = 3 donors, data presented as mean ± s.e.m.), and correlation of GITR and PD-1 mRNA-seq expression in live cells (**c**) (*n* = 21 tumors) and T cells (**d**) (*n* = 18 tumors) sorted from HNSCC tumor samples. Statistical significance was calculated with nonlinear regression. Live and T cells, *P* = 0.00001 and *P* = 0.067, respectively. **e**, Representative CD8, PD-1, GITR and FoxP3 multiplex immunofluorescence imaging (excluding PanCK, CD3 and DAPI) and spatial distribution cell density heatmaps (cells mm^–2^) (CD8, PD-1 and GITR) of FFPE sections of primary HNSCC (excluded compartment) and matching lymph node metastases (LN mets) (20×). Selected areas of GITR^+^PD-1^+^CD8^+^ T cells are indicated in white boxes. Experiment is representative of *n* = 3 tumor samples. **f**,**g**, Schematic diagram of anti-PD-1–GITR-L bispecific (**f**) and an example of a 2D class average based on negative-stain TEM of the whole molecule (**g**). **h**,**i**, Binding of anti-huPD-1-huGITR-L to huPD-1- (**h**) and GITR-transfected (**i**) HEK293S cells (*n* = 2 technical cell culture replicates within a single experiment). **j**,**k**, Binding of anti-huPD-1-huGITR-L to human CD4^+^CD45RA^–^CCR7^+^ central memory T cells (T_CM_) (**j**) and CD4^+^CD45RA^–^CCR7^–^ effector memory T cells (T_EM_) (**k**) (*n* = 2 donors). **l**,**m**, GITR-NFκB (**l**) and PD-1/PD-L1 NFAT signaling (**m**) on HEK293-NFκB-huGITR^+^ and Jurkat-NFAT-huPD-1^+^ (with CHO-K-PD-L1^+^ cells) reporter cells following threefold titration of the anti-huPD-1-huGITR**-**L bispecific (*n* = 2 technical cell culture replicates within a single experiment). MFI, median fluorescence intensity. RLU, relative luminescence units. Source data

**Extended Data Fig. 2:**
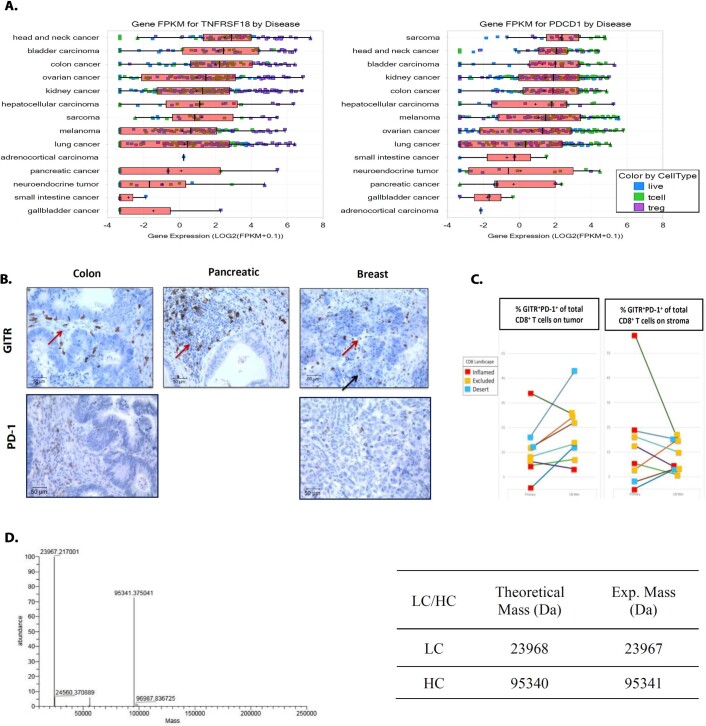
Human PD-1 and GITR expression on TILs by mRNA-seq and IHC and LC-MS of anti-huPD-1-huGITR-Lbispecific. (**A**) Gene mRNA expression of GITR and PD-1 across different tumor types (UCSF-IPI, n = 50 tumors). For box-and-whiskers plots, boxes represent 25^th^ and 75^th^ percentiles, center lines indicate median values and whiskers represent minimum and maximum values. (**B**) Representative human PD-1 and GITR expression by IHC on colon, pancreatic and breast cancer tissues. Frozen tissue microarray samples were used for PD-1 and GITR staining. Scale bars indicate 50 µm (original magnification 40x, representative staining of n = 5 tissues). (**C**) Percentage of GITR^+^PD-1^+^ cells of total CD8^+^ T cells within inflamed, excluded and desert compartments of HNSCC including the tumor, stroma, and tumor-proximal lymph node aggregates (n = 8 samples) (**D**) LC-MS of reduced and de-glycosylated anti-huPD-1-huGITR-L bispecific (hIgG1-LALA) with theoretical and experimental masses of light (LC) and heavy chain (HC).

PD-1 and GITR coexpression has been demonstrated on recently T-cell receptor (TCR)-activated T cells from peripheral blood mononuclear cells (PBMCs) by flow cytometry (Fig. 2a,b). High PD-1 and GITR expression levels have been also found in tumor-infiltrating lymphocytes among different cancer indications based on messenger RNA sequencing (mRNA-seq) analysis (University of California at San Francisco (UCSF) immunoprofiler initiative) and immunohistochemistry (IHC), as previously suggested (Extended Data Fig. 2a,b and Supplementary Table 1)^24^. A high correlation of GITR and PD-1 gene expression has been found in live T cells sorted from head and neck squamous cell carcinoma (HNSCC) (Fig. 2c,d). Spatial distribution imaging analysis (multiplex immunofluorescence) suggested that a small fraction of CD8^+^PD-1^+^ T cells are also GITR^+^ (FoxP3^–^) within inflamed and excluded compartments of HNSCC, including the tumor, stroma and tumor-proximal lymph node aggregates (Fig. 2e and Extended Data Fig. 2c).

### Characterization of the human anti-PD-1–GITR-L bispecific

The bispecific is an anti-huPD-1 antibody (clone 12A11) fused via flexible (Gly_3_Ser)_4_ linkers to the N terminus of extracellular domains of two human GITR-L trimers connected by (Gly_3_Ser)_4_ linkers. The Fc portion of the bispecific lacks FcγR-mediated effector functions (hIgG1-LALA) (Fig. 2f)^25^. Structural analysis by negative-stain transmission electron microscopy (TEM) showed different conformations of the bispecific, which indicates structural flexibility (Fig. 2g). The predicted mass of the bispecific (~242 kDa) was confirmed by liquid chromatography–mass spectrometry (Extended Data Fig. 2d).

**Extended Data Fig. 3:**
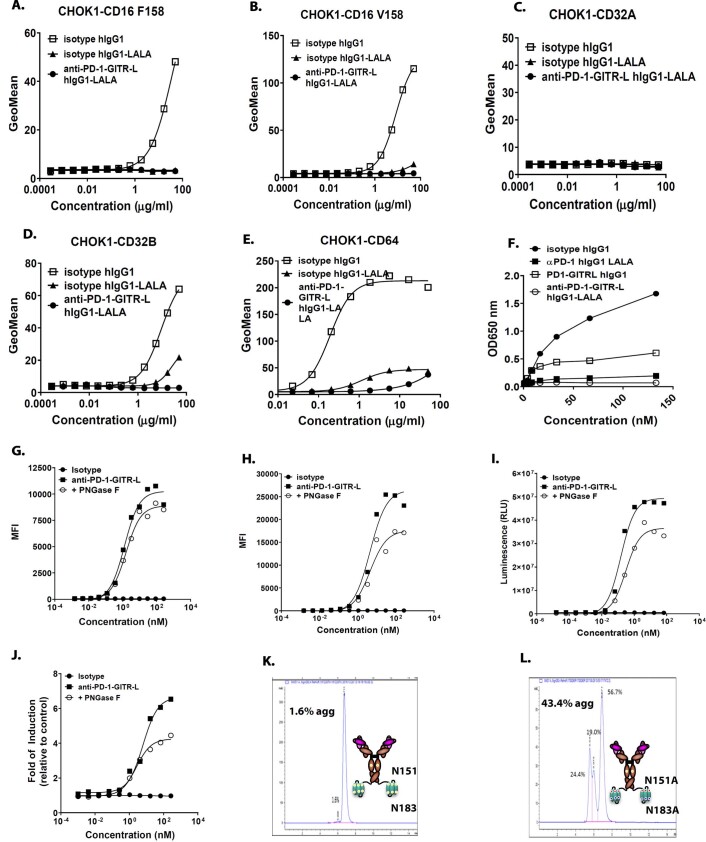
Binding of anti-PD-1-GITR-L-hIgG1-LALA bispecific to human FcγR-CHO stable transfectants/complement (C1q) and binding/in vitro bioactivity of anti-PD-1-GITR-L bispecific following de-glycosylation with PNGase F. (**A** to **F**) Binding of anti-PD-1-GITR-L-hIgG1-LALA bispecific, and controls to human FcγR-CHO stable transfectants (**A** to **E**) (representative data of n = 2 independent experiments), and human C1q (**F**) (n = 2 technical cell culture replicates within a single experiment). **(G** to **J)** Binding of de-glycosylated variants of anti-huPD-1-huGITR-L to human PD-1 (**G**) and GITR (**H**) transfected HEK293S cells (n = 2 technical cell culture replicates within a single experiment), and *in vitro* bioactivity (GITR-NFκB and PD-1/PD-L1 NFAT signaling on HEK293-NFκB-huGITR^+^ and Jurkat-NFAT-huPD-1^+^ reporter cells) (n = 2 technical cell culture replicates within a single experiment) (**I** and **J**). (**K** and **L**) Size exclusion chromatograms of N151-N183 and N151A-N183A anti-PD-1-GITR-L mutants. Source data

Human target cell-surface binding affinity, kinetics and signaling of anti-huPD-1-huGITR-L were conserved on HEK293 cells, human PBMCs and signaling reporter assays (Fig. 2h–m and Supplementary Tables 2 and 3). FcRn and β_2_m binding of anti-huPD-1-huGITR-L were conserved at pH 6.0, and no binding to human FcγRs or C1q was detected (Supplementary Table 4 and Extended Data Fig. 3a–f).

Total glycan analysis of anti-huPD-1-huGITR-L demonstrated that the Fc N-glycosylation site (N297) was glycosylated and that each of the monomeric huGITR-L subdomains contained two N-glycosylation sites (N151 and N183). Based on structural data, N151 has been localized at the top rim of the trimeric huGITR-L conformational assembly while N183 was found close to the ligand–CRD2 receptor interface. Partial deglycosylation resulted in partial loss of binding affinity and bioactivity (Extended Data Fig. 3g–j and Supplementary Table 5). Replacing the N151 and N183 residues of the huGITR-L domain of the bispecific with alanine resulted in a higher degree of aggregation, suggesting that both N-glycosylation sites may play a role in trimeric core packing of the GITR-L subunits and in stabilization of the ligand–receptor interaction, due to limited hydrophobic residues (Extended Data Fig. 3k,l). Based on these findings, N-glycosylation sites remain unmutated in the ligand domains of anti-huPD-1-huGITR-L.

### Anti-muPD-1-muGITR-L induces an immunologically driven mechanism

The surrogate anti-muPD-1-muGITR-L is an equivalent form of the human bispecific (anti-PD-1 clone 17D2). A dimeric murine ligand was used for the surrogate bispecific based on previous structural data^26,27^. Similar to the human bispecific, the mouse surrogate also has null effector functions (mIgG2a-DANA, D265A-N297A). PD-1 and GITR binding affinity of anti-muPD-1-muGITR-L was equivalent to that of anti-muPD-1 and isotype-muGITR-L (Supplementary Table 5).

**Fig. 3:**
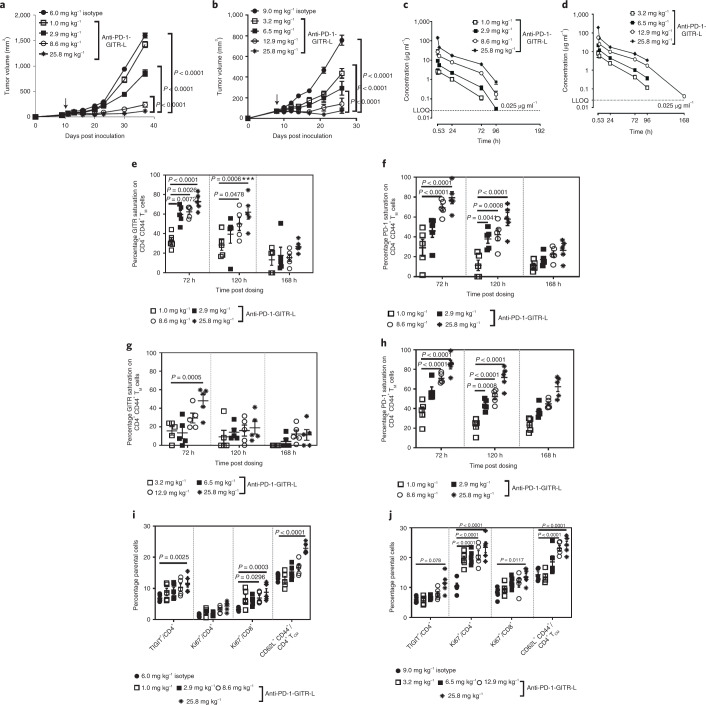
The anti-muPD-1-muGITR-L bispecific induces dose-dependent growth inhibition and peripheral target engagement, T cell activation and proliferation in CT26 and EMT6 tumor-bearing mice. **a**,**b**, Growth inhibition of CT26 and EMT6 cells in syngeneic mice by anti-muPD-muGITR-L. Titration of the bispecific at indicated doses was administered IV following one dose in CT26 (**a**) and EMT6 (**b**), respectively. Each point on the curve represents mean tumor volume for each group (*n* = 10 mice). **c**,**d**, Serum concentration versus time profile following IV administration of the bispecific at the indicated doses in CT26 (**c**) and EMT6 (**d**) (*n* = 4 mice). **e**–**j**, Flow cytometry analysis of blood lymphocytes after treatment with anti-muPD-1-muGITR-L. Blood samples were collected from CT26 and EMT6 tumor-bearing mice at the indicated times following either treatment with isotype control or titration of anti-muPD-muGITR-L administered IV following one dose, at the indicated doses. CD4^+^ T cells were assessed for percentage of GITR (CT26 (**e**) and EMT6 (**g**)) and PD-1 (CT26 (**f**) and EMT6 (**h**)) expression. Percentage target saturation was standardized to 0% at *t* = 0. TIGIT^+^, CD62^–^CD44^+^ T_CM_ and Ki67^+^ are shown as a percentage of CD4^+^ while Ki67^+^ is shown as a percentage of CD8^+^ T cells in the blood (CT26 (**i**) and EMT6 (**j**)). Results for five animals per group from a single experiment were averaged, and standard deviations are shown, Statistical significance was calculated using two-way ANOVA with Tukey’s correction for multiple comparisons (statistics refer to anti-PD-1–GITR-L bispecific (25.8, 8.6 or 12.9, 2.9 or 6.5 mg kg^–1^) versus isotype control). LLOQ, lower level of quantitation. Source data

**Extended Data Fig. 4:**
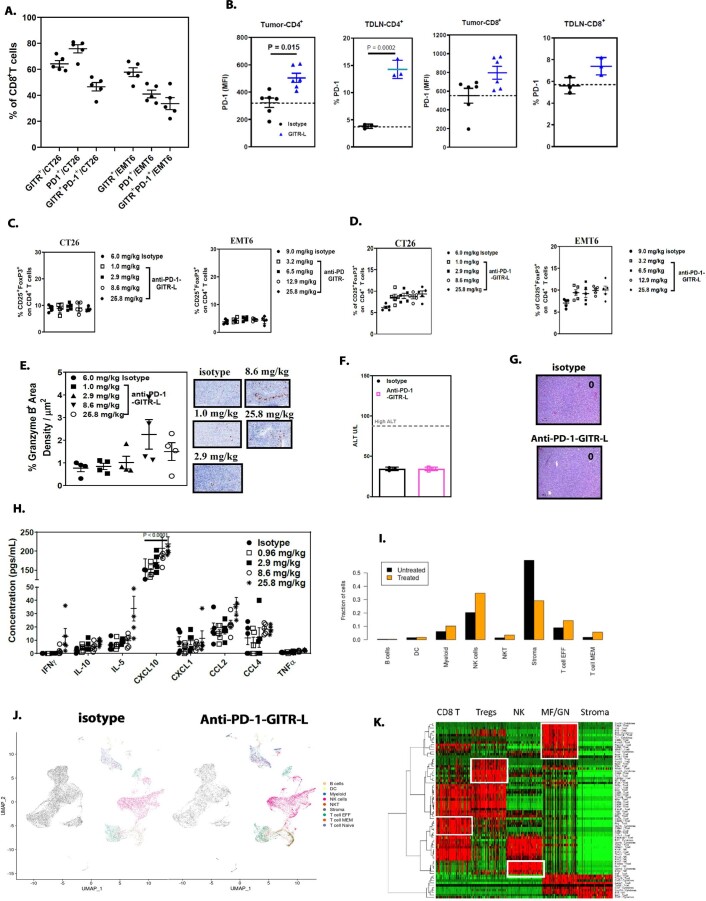
Expression of GITR and PD-1 on mouse T cells before/after anti-muPD-1-muGITR-L dosing, toxicity following dosing of anti-muPD-1-muGITR-L in CT26 model, and single-cell mRNAseq analysis of CD45 TILs. (**A**) Flow cytometry analysis of GITR and PD-1 expression on tumor infiltrated CD8^+^ T cells collected from CT26 and EMT6 models (n = 5 mice). (**B**) Flow cytometry analysis of PD-1 expression on tumor (n = 5 per group) and TDLN (n = 3 per group) infiltrated CD4^+^ and CD8^+^ T cells following treatment of CT26 model with isotype-GITR-L construct (1.43 mg/kg, 3X, ip, tissues collected 24 hrs after 3rd dose). Statistical significance was calculated with two-way ANOVA with Tukey’s correction for multiple comparisons (statistics refer to isotype-GITR-L vs isotype control). Flow cytometry analysis of blood (**C**) and TDLN (**D**) Treg cells following treatment with anti-muPD-1-muGITR-L in CT26 and EMT6 tumor bearing mice 120 hours post dose (n = 5 per group). CD25^+^FoxP3^+^ are shown as a percent of CD4^+^ T cells. Data are presented as mean values +/- SEM. (**E**) anti-muPD-muGITR-L dose-dependent increase of GZB^+^ (HALO imaging analysis) intra-tumoral immune cells by IHC in CT26 model (7 days after one dose). Representative GZB tumor IHC staining from selected mice is also shown. Scale bars indicate 200 µm (original magnification 20x) (n = 4 per group). Data are presented as mean values +/- SEM. (**F**) Serum ALT level in treated mice at efficacious dose (i.p. 3Qw for 1 week at 4.3 mg/kg) in CT26 model (n = 3 per group). Data are presented as mean values +/- SEM (**G**) Representative H&E IHC (representative staining of n = 3 tissues) of liver samples (FFPE). Serum and liver samples were collected 1 day after third dose. Scale bars indicate 50 µm (original magnification 20x). (**H**) Plasma level of cytokines and chemokines collected 72 hours after one dose of anti-PD-1-GITR-L bispecific (n = 5 per group). Data are presented as mean values +/- SEM. Statistical significance was calculated with two-way ANOVA with Tukey’s correction for multiple comparisons (statistics refer to anti-PD-1-GITR-L at 25.8 mg/kg vs isotype control). (**I** to **K**) Single-cell mRNA-seq analysis of CD45^+^ enriched tumor infiltrating lymphocytes following treatment with anti-muPD-1-muGITR-L bispecific (25.8 mg/kg) vs isotype in the CT26 model. Tumors were collected 5 days following treatment. (**I**) UMAP plots align immune cell subset clusters comparing effect of isotype vs anti-muPD-1-muGITR-L bispecific. (**J**) Bar plot shows the percentages of immune cell subset clusters. (**K**) Unique gene expression profile of CD8+ T, NK, Treg, myeloid and stroma cell populations (white square). Source data

Dose-dependent antitumor efficacy was observed following one dose of anti-muPD-1-muGITR-L in mice bearing subcutaneous (SC) syngeneic CT26 or EMT6 tumors (Fig. 3a,b). A noncompartmental pharmacokinetic (PK) analysis of the bispecific showed a linear PK profile with a half-life ranging from 14 to 17 h for CT26, and from 17 to 32 h for EMT6. Both maximum concentration (*C*_max_) and area under the curve (AUC) from zero to infinity (AUC_inf_) increased in a dose-proportional manner (Fig. 3c,d and Supplementary Table 6). Blood samples were collected from CT26 and EMT6 models following one dose of anti-muPD-1-muGITR-L, for analysis of target saturation and bioactivity. Anti-muPD-1-muGITR-L treatment resulted in dose-dependent partial saturation of GITR and PD-1 on CD4^+^ T cells in the circulation. GITR and PD-1 desaturation was observed at 120 and 168 h post dose, respectively (Fig. 3e–h). Differences observed in PK and target desaturation in the circulation are probably due to slightly different levels of target expression on T cells in these two models, as shown for CD8^+^ T cells within the tumor microenvironment (Extended Data Fig. 4a), and due to upregulation of GITR-L-mediated PD-1 expression (Extended Data Fig. 4b).

**Fig. 4:**
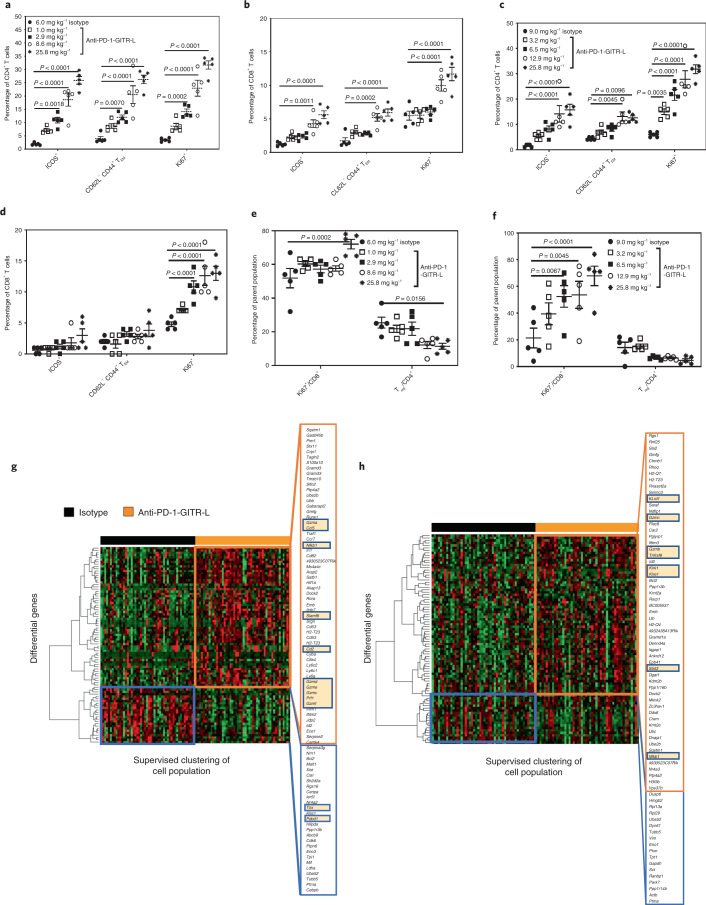
The anti-muPD-muGITR-L bispecific induces increased activation and proliferation of TDLNs and intratumoral T and NK cells in CT26 and EMT6 tumor-bearing mice. **a**–**f**, Flow cytometry analysis of TDLNs and TiLs after treatment with anti-muPD-muGITR-L. Draining lymph nodes and tumors were collected from CT26 and EMT6 tumor-bearing mice 120 h following treatment with isotype control or a titration of anti-muPD-muGITR-L administered IV following one dose at the indicated doses. ICOS^+^, CD62L^–^CD44^+^ T_EM_ and Ki67^+^ are shown as a percentage of CD4^+^ (**a**) and CD8^+^ T cells (**b**) in CT26 TDLNs. ICOS^+^, CD62L^−^CD44^+^ T_EM_ and Ki67^+^ are shown as a percentage of CD4^+^ (**c**) and CD8^+^ T cells (**d**) in EMT6 TDLNs. Ki67^+^ is shown as a percentage of CD8^+^ T cells and CD25^+^FoxP3^+^ as a percentage of CD4^+^ T cells in the tumor (CT26 (**e**) and EMT6 (**f**)). **a**–**f**, Results for five animals per group were averaged, and standard deviations are shown. Statistical significance was calculated by two-way ANOVA with Tukey’s correction for multiple comparisons (statistics refer to the anti-PD-1–GITR-L bispecific (25.8, 8.6 or 12.9, 2.9 or 6.5 mg kg^–1^) versus isotype control). **g**,**h**, Single-cell mRNA-seq analysis of CD45^+^-enriched TiLs following treatment with the anti-muPD-1-muGITR-L bispecific (25.8 mg kg^–1^) versus the isotype in the CT26 model. Tumors were collected 5 days following treatment. Supervised clustering of CD8^+^ T (**g**) and NK (**h**) cells following treatment with the anti-muPD-1-muGITR-L bispecific versus isotype control (orange rectangle, upregulated genes; blue rectangle, downregulated genes). Specific genes are indicated by yellow highlighting (*n* = 2 mice). Source data

Anti-muPD-1-muGITR-L treatment resulted in a dose-dependent increase in the percentages of TIGIT^+^CD4^+^ T cells, CD62L^+^CD44^+^CD4^+^ T_CM_ and Ki67^+^ CD4^+^ and CD8^+^ T cells in the circulation 168 h after dosage (Fig. 3i,j). A dose-dependent increase in the percentages of ICOS^+^, CD62L^–^CD44^+^ T_EM_ and Ki67^+^ in CD4^+^ and CD8^+^ T cells was observed at 120 h in tumor-draining lymph nodes (TDLNs), while tumor-infiltrated lymphocytes (TILs) showed a dose-dependent increase in the percentage of CD8^+^Ki67^+^ T cells, a decrease in the percentage of CD4^+^CD25^+^Foxp3^+^ cells (T_regs_) and an apparent increase in granzyme B^+^ (GZMB) (Fig. 4a,b,e for CT26, Fig. 4c,d,f for EMT6 and Extended Data Fig. 4e for CT26). Non notable effects were observed in T_reg_ cells within the circulation and TDLNs (Extended Data Fig. 4c,d). No evidence of liver toxicity or acute cytokine release syndrome was observed in bispecific-treated CT26 mice (Extended Data Fig. 4f–h). Only CXCL10 was detected, and this is considered a soluble biomarker of cytotoxic CD8^+^ T cell trafficking.

**Extended Data Fig. 5:**
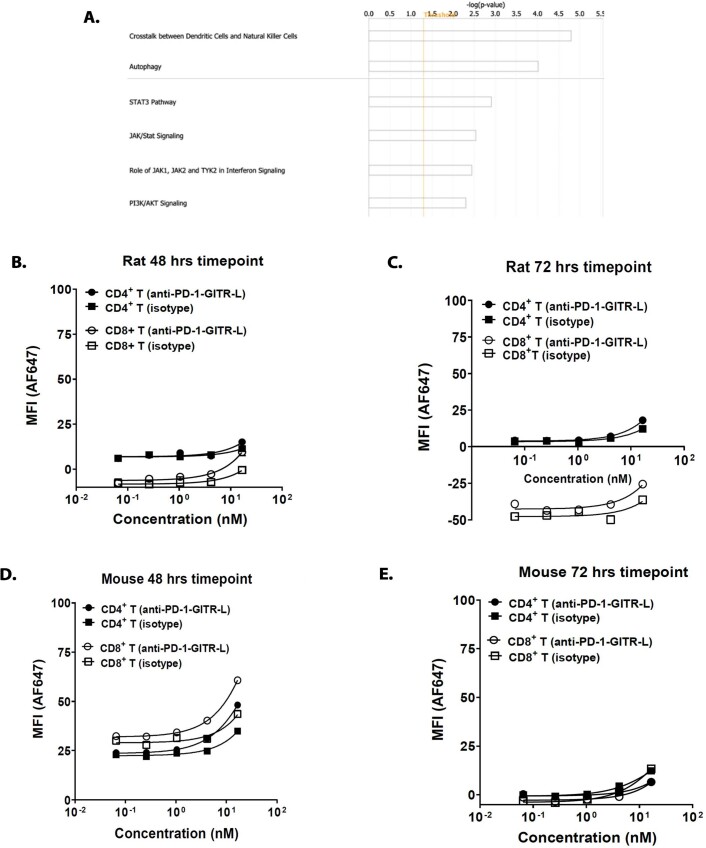
Downstream pathway analysis following dosing of anti-muPD-1-muGITR-L bispecific in CT26 tumor syngeneic model, and rat/mouse cross reactivity of anti-huPD-1-huGITR-L bispecific. (**A**) Gene ontology biological processes (GO-BP) enrichment analysis of intratumoral CD8^+^ T and NK cells between isotype and anti-PD-1-GITR-L-treated mice shows enriched pathways (hypergeometric test, adjusted P-values obtained by Benjamini-Hochberg procedure). (**B** to **E**) Binding of Alexa fluor (AF) 647-labeled anti-huPD-1-huGITR-L to anti-CD3 activated rat (**B** and **C**) and mouse splenocytes (**D** and **E**) (t = 48 and 72 hrs). Two independent experiments were performed. Source data

In addition, single-cell mRNA-seq analysis of tumors showed that anti-muPD-1-muGITR-L induced an increase in the number of CD8^+^ T_eff_ and NK cells in the CT26 model, based on immune cell subset clustering analysis (Extended Data Fig. 4i,j). Immune cell gene markers showed distinct expression profiles in different types of immune cells (Extended Data Fig. 4k). Annotation of immune cell subtypes was based on an analysis of the Immunological Genome Project database (ImmGen)^28^. Supervised clustering analysis revealed an enrichment of CD8^+^ T and NK cells with a differentiated expression profile following treatment with anti-muPD-1-muGITR-L in comparison to the isotype control, which displayed higher levels of *NFκB1*, *STAT3*, *KLRE1*, *KLRD1*, *KLRK1*, *TNFRSF9*, *CCL5*, *CD2*, *GZMC*, *GZMB*, *GZMA*, *GZMD*, *GZME*, *GZMF* and *PRF1*, with upregulation of genes involved in activation, survival, homeostasis and interferon- and cytokine-related signaling pathways (Figs. 4g,h and Extended Data Fig. 5a). An increase in the progenitor gene marker *SLAMF6* and a decrease in exhaustion gene markers *TOX* and *PD-1* were also observed for CD8^+^ T cells^29^. Therefore, optimal efficacy in these models correlated with an increase in activated, memory and proliferating CD4^+^ and CD8^+^ T cells in the blood, TDLNs and tumor, an increase in cytotoxic NK cells and a reduction in T_reg_ and exhausted CD8^+^ T cells in the tumor microenvironment (TME).

### Anti-huPD-1-huGITR-L is active in both engineered and humanized models

**Fig. 5:**
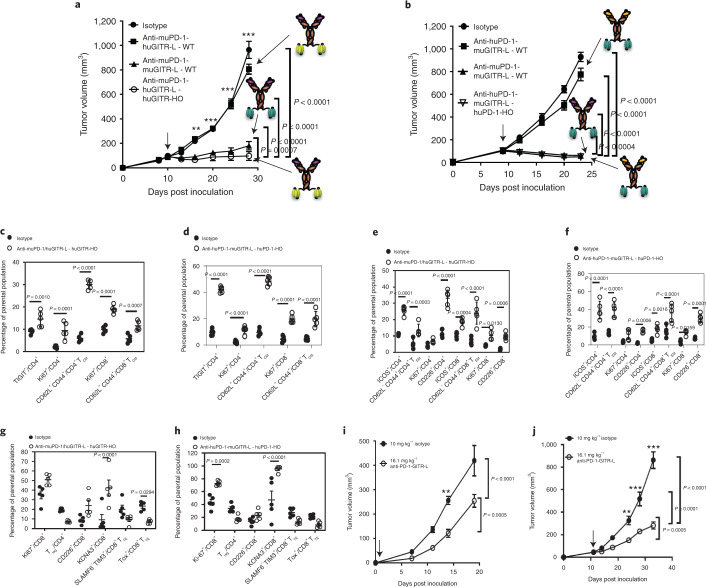
The anti-huPD-1-huGITR-L bispecific induces MC-38 tumor growth inhibition in genetically engineered and humanized mouse models. **a**,**b**, Growth inhibition of MC-38 cells by anti-muPD-1-huGITR-L (**a**) and anti-huPD-1-muGITR-L chimeric bispecific (**b**) in human GITR and PD-1 transgenic models in comparison to anti-muPD-muGITR-L surrogate bispecific in WT mice. Bispecific domains are indicated by the following colors: purple, variable domain of anti-muPD-1; green, muGITR-L; yellow, huGITR-L; and orange, variable domain of anti-huPD-1. Mice were treated with one dose of isotype at 1.0 mg kg^–1^, surrogate bispecific at 1.3 mg kg^–1^ and chimeric bispecific at 1.46 mg kg^–1^ (*n* = 8 mice). **c**,**d**, TIGIT^+^, CD62L^−^CD44^+^ T_CM_ and Ki67^+^ are shown as a percentage of CD4^+^ in blood while CD62L^−^CD44^+^ T_CM_ and Ki67^+^ are shown as a percentage of CD8^+^ T cells following treatment with anti-muPD-1-huGITR-L in huGITR homozygous (HO) mice (**c**) and anti-huPD-1-muGITR-L in huPD-1 HO mice (**d**). **e**,**f**, ICOS^+^, CD62L^−^CD44^+^ T_EM_, Ki67^+^ and CD226^+^ are shown as a percentage of CD4^+^ and CD8^+^ T cells in TDLN following treatment with anti-muPD-1-huGITR-L in huGITR HO mice (**e**) and anti-huPD-1-muGITR-L in huPD-1 HO mice (**f**). **g**,**h**, Ki67^+^, CD226^+^, KCNA3^+^, SLAMF6^−^TIM3^+^ and TOX^+^ are shown as a percentage of CD8^+^ T cells in the tumor, and CD25^+^FoxP3^+^ as a percentage of CD4^+^ T cells following treatment with anti-muPD-1-huGITR-L in huGITR HO mice (**g**) and anti-huPD-1-muGITR-L in huPD-1 HO mice (**h**). Tissues were collected 120 h after treatment. Results from five animals per group were averaged, and standard deviations are shown. **i**,**j**, Growth inhibition of xenograft PC-3 (**i**) and HCT-116 (**j**) cells in NSG allogeneic PBMC-reconstituted mice following treatment with one dose of anti-huPD-1-huGITR-L at the indicated doses. Each point on the curve represents mean ± s.e.m. of tumor volume for each group (*n* = 8 per group in transgenic HO huPD-1 and huGITR and PC-3 mouse models, and *n* = 10 per group in HCT-116 mouse model). Statistical significance was calculated by two-way ANOVA with Tukey’s correction for multiple comparisons (statistics refer to chimeric anti-PD-1–GITR-L bispecific versus isotype control). **P = 0.0005, ***P = 0.0001. Source data

**Extended Data Fig. 6:**
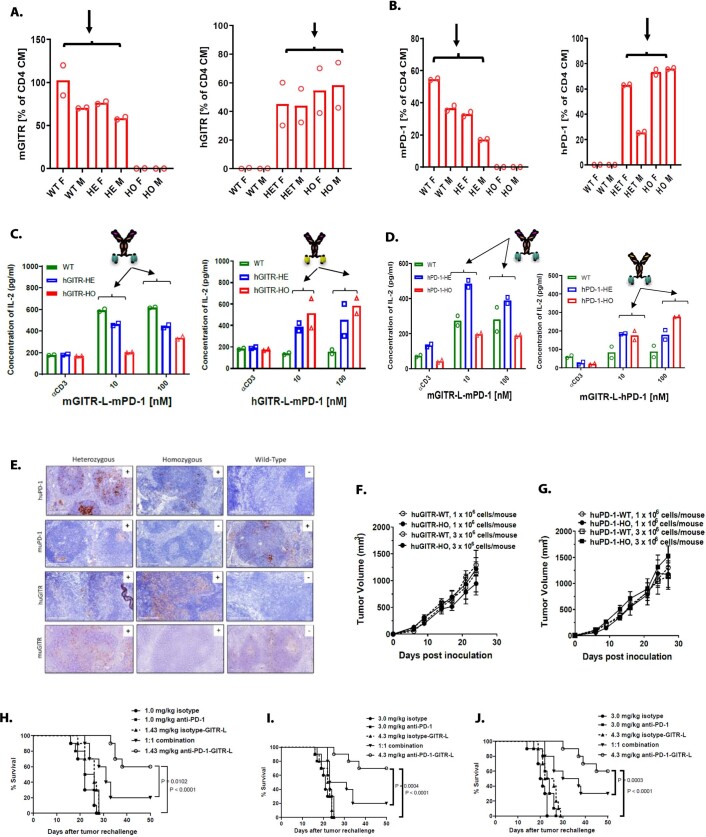
Expression/in vitro/in vivo characterization of GITR and PD-1 in heterozygous and homozygous GEM, and anti-muPD-1-muGITR-L enhanced survival of CT26, EMT6 and JC tumor bearing mice in comparison to monotherapies and combination. (**A** and **B**) Mouse and human target expression on anti-CD3 activated CD4^+^CD44^+^CD62L^+^ T cells isolated from spleens of indicated mice (A. n = 2 & B. n = 2). (**C** and **D**) IL-2 secretion following treatment with mouse surrogate and chimeric bispecifics (C: mGITR-L-mPD-1 and hGITR-L-mPD-1, and D: mGITR-L-mPD-1 and mGITR-L-hPD-1) using anti-CD3 activated splenocytes isolated from indicated mice. Color of bispecific domains indicated: purple = variable domain of anti-muPD-1, green = muGITR-L, yellow = huGITR-L and orange = variable domain of anti-huPD-1. A single experiment was conducted with 2 replicates and mean values were plotted. (**E**) Representative human and Mouse PD-1 and GITR expression by IHC (representative staining of n = 4 tissues) on spleens isolated from indicated mice. Scale bars indicate 100 µm (original magnification 20x). (**F** and **G**) Tumor baseline study of MC-38 cell line in huGITR (F) and huPD-1 (**G**) homozygous vs wild-type C57BL/6 mice following inoculation of 1 and 3 × 10^6^ cells/mouse. Each point on the curve represents the mean tumor volume for each group (n = 5 mice per group for huGITR & n = 7 mice per group for hu PD-1). Data are presented as mean values +/- SEM. (**H** to **J**) Mice survival following dosing of anti-muPD-1-muGITR-L bispecific. Results depict cumulative survival curves with indicated treatments: (**H**) CT26, (**I**) EMT6 and (**J**) JC models (n = 10 mice per group, ~ 100 mm^3^ tumor at time of dosing). Statistical significance was calculated with log-rank test with post-hoc analysis for multiple comparisons (statistics refer to anti-PD-1-GITR-L vs combo). Source data

The dvelopment of humanized target knock-in (KI) mouse tumor syngeneic models has been shown to be instrumental in evaluation of the in vivo efficacy of anti-huCTLA-4 and anti-PD-1 antibodies^30,31^. The antitumor efficacy of chimeric bispecifics (anti-muPD-1-huGITR-L and anti-huPD-1-muGITR-L) was tested in single KI human GITR and PD-1 homozygous genetically engineered mouse models. Transgenic mice were used due to lack of binding of anti-huPD-1-huGITR-L to activated rat and mouse T cells (Extended Data Fig. 5b–e). Human target expression and absence of mouse target expression was confirmed on activated central memory T cells isolated from the spleen of homozygous mice (Extended Data Fig. 6a,b). The chimeric bispecifics induced an increase in IL-2 secretion in splenocytes isolated from both homozygous and heterozygous mice (Extended Data Fig. 6c,d). In comparison, minimal bioactivity was observed with the surrogate bispecific in homozygous mice, and with the chimeric bispecifics in splenocytes isolated from wild-type (WT) mice. IHC staining studies confirmed human target expression in spleen and lymph nodes isolated from homozygous mice (Supplementary Table 7 and Extended Data Fig. 6e). A similar take-up and growth rate in the MC-38 tumor cell line was observed in both transgenic homozygous models in comparison to WT mice (Extended Data Fig. 6f,g). Bioactivity results were confirmed in vivo by induction of MC-38 tumor growth inhibition following treatment with the chimeric bispecifics in homozygous transgenic mice (Fig. 5a,b). Similar antitumor efficacy was observed with surrogate anti-muPD-1-muGITR-L in WT mice. No notable efficacy was seen in WT mice following treatment with the chimeric bispecifics, which confirms that coengagement of PD-1 and GITR is critical for the activity of anti-PD-1–GITR-L. Also, chimeric bispecifics induced an increase in the percentage of TIGIT^+^CD4^+^, Ki67^+^ and T_CM_ CD4^+^ and CD8^+^ T cells from blood, and ICOS^+^, CD62L^–^CD44^+^T_EM_, Ki67^+^ and CD226^+^ in CD4^+^ and CD8^+^ T cells from TDLNs of homozygous mice (Fig. 5c–f). In tumors we observed an increasing trend for Ki67^+^, CD226^+^ and KCNA3^+^ in CD8^+^ T cells, and a decreasing trend for T_regs_, SLAMF6^–^TIM3^+^ and TOX^+^ terminally exhausted CD8^+^ T (T_TE_) cells (Fig. 5g,h). SLAMF6 has been identified as a cell-surface marker (equivalent to TCF-1) that distinguishes progenitor exhausted (T_PE_) from T_TE_ antigen-specific CD8^+^ TILs^32^. In conclusion, the bioactivity of chimeric anti-PD-1–GITR-L constructs has been demonstrated only in humanized single-target KI mouse tumor models, with no sign of bioactivity in WT mice, which not only validates PD biomarkers observed with the surrogate bispecific in WT mice but clearly suggests that target coengagement is crucial for the mechanism of action (MoA) of the bispecific.

Humanized mouse models have previously been used to test the bioactivity of immunotherapies in xenograft models^33–36^. The efficacy of anti-huPD-1-huGITR-L was also tested in non-obese diabetic scid gamma (NSG) humanized mouse xenograft tumor models (PC-3 and HCT-116) following engraftment with allogeneic human T cells and monocyte-derived dendritic cells (moDCs). Compared to isotype control monoclonal antibody (mAb), a single dose of anti-huPD-1-huGITR-L significantly inhibited growth of human tumor cell lines, either at inoculation or after tumor establishment following a dose of 16.1 mg kg^–1^ (Fig. 5i,j).

### Anti-PD-1–GITR-L has a different MoA than the combination

**Fig. 6:**
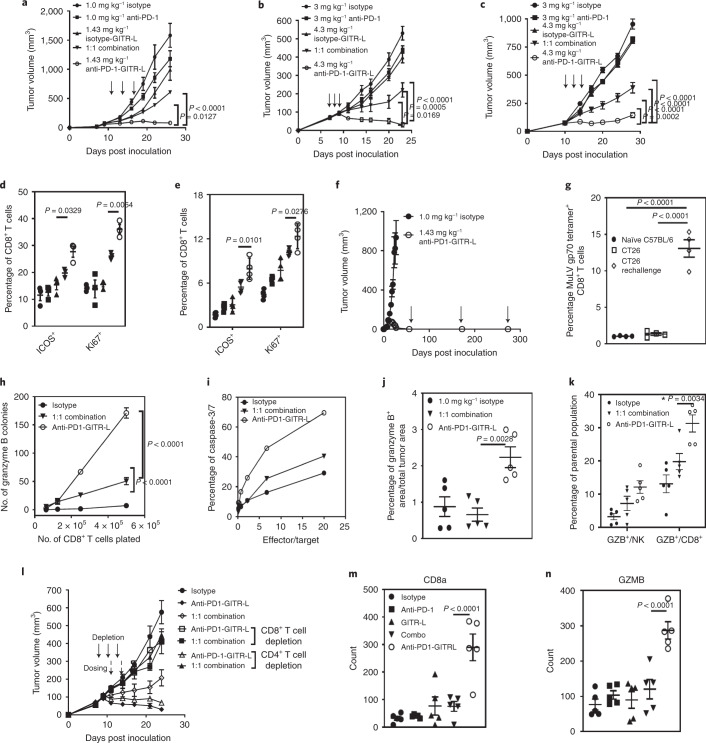
The anti-muPD-1-muGITR-L bispecific has different bioactivity in vivo in comparison to anti-muPD-1 plus muGITR-L combination and monotherapies in anti-PD-1 resistant tumor syngeneic models. **a**–**c**, Growth inhibition of CT26 (**a**), EMT6 (**b**) and JC cells (**c**) in syngenic mice following indicated treatments and doses (IP frequency indicated by arrows). Each point on the curve represents the mean tumor volume for each group (*n* = 7 mice for CT26 and *n* = 10 for EMT6 and JC). **d**,**e**, Flow cytometry analysis of draining lymph nodes collected from CT26 and JC models (24 h post second dose). ICOS^+^ and Ki67^+^ are shown as a percentage of CD8^+^ T cells (CT26 (**d**) (*n* = 3 mice) and JC (**e**) (*n* = 4 mice)). **f**,**g**, CT26 tumor growth rechallenge study (**f**) (*n* = 7 mice) and accumulation of MuLV gp70-antigen-specific T cells (**g**) in a fully regressed CT26 model following treatment with anti-muPD-muGITR-L bispecific (*n* = 4 mice). **h**–**k**, Number of CT26-specific GZMB^+^ CD8^+^ T cells (TDLNs (**h**) (*n* = 3 mice)), percentage of CT26 cell killing measured by caspase-3/7 staining (**i**), percentage of CT-26 specific GZMB^+^ CD8^+^ T cells (TILS (**j**) (*n* = 5 mice)) and GZMB^+^ shown as percentage of CD3^−^CD49b^+^ NK and CD8^+^ T cells in the tumor (**k**) (*n* = 5 mice). **l**, Growth inhibition of EMT6 cells in syngeneic mice by anti-muPD-muGITR-L bispecific and 1:1 combination following in vivo depletion of CD8^+^ and CD4^+^ T cells (*n* = 10 mice). **m**,**n**, Tumor NanoString analysis of CD8a (**m**) and GZMB genes (**n**) (CT26 model, *n* = 5 mice). Each point on the curve represents the mean tumor volume for each group. **a**–**h**, **j**–**n**, Data presented as mean ± s.e.m. Statistical significance was calculated by two-way ANOVA with Tukey’s correction for multiple comparisons (statistics refer to anti-PD-1–GITR-L bispecific versus combination). Source data

**Extended Data Fig. 7:**
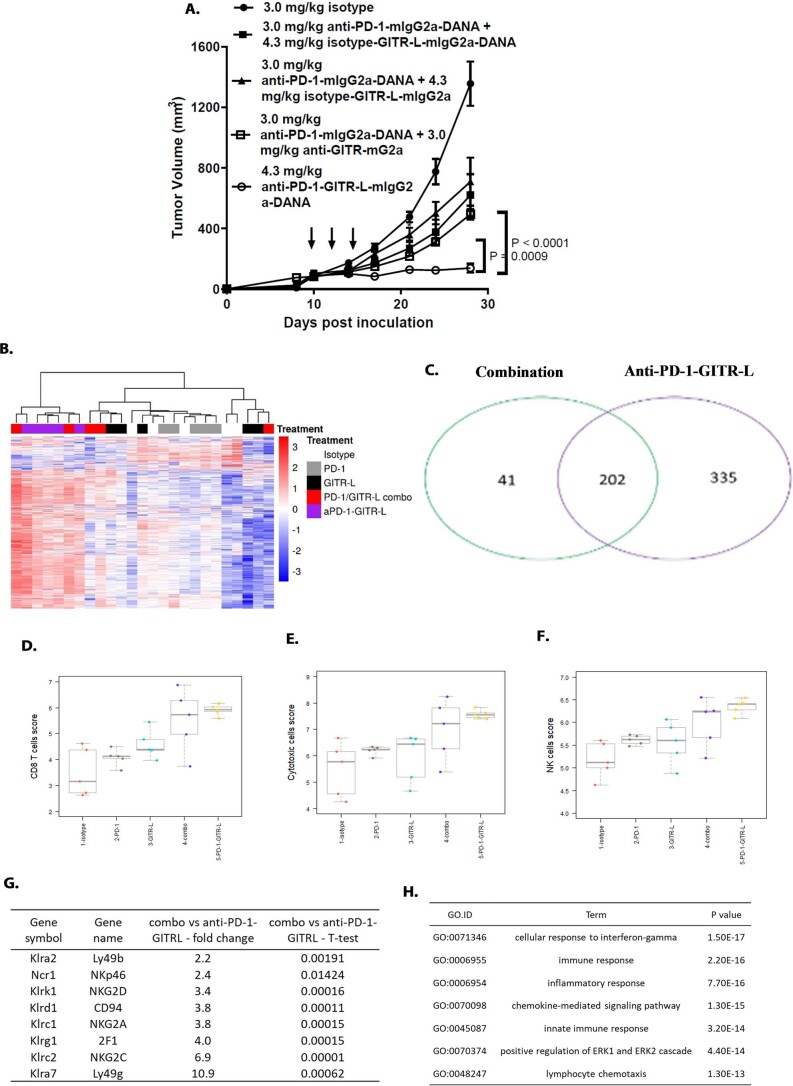
The anti-muPD-1-muGITR-L-mIgG2a-DANA bispecific has different bioactivity in vivo in comparison to the combination of anti-PD-1 (mIgGa-DANA) and isotype-GITR-L (mIgG2a-DANA or mIgG2a) or anti-GITR (mIgG2a) in JC tumor syngeneic models, and anti-muPD-1-muGITR-L induced a higher propensity for immune activity within tumors than the combination and monotherapies. (**A**) Growth inhibition of JC tumor model following indicated treatments and doses (i.p. frequency indicated by arrows). Each point on the curve represents the mean tumor volume for each group (n = 7 mice). Data are presented as mean values +/- SEM. Statistical significance was calculated with two-way ANOVA with Tukey’s correction for multiple comparisons (statistics refer to nti-PD-1-GITR-L vs anti-PD-1 plus anti-GITR-mIgG2a). (**B**) Tumor Nanostring gene clustering analysis. Heatmap was generated by R package pheatmap (version 1.0.12), and (**C**) number of intra-tumoral altered genes (> 2-fold) following treatment with the combination vs anti-muPD-1-muGITR-L bispecific in the CT26 model. (**D** to **F**) Tumor CD8^+^ T (D), cytotoxicity (**E**) and NK cell (**F**) Nanostring gene signature quantification analysis following treatment of CT26 with indicated treatments (n = 5 mice). For box-and-whiskers plots, boxes represent 25th and 75th percentiles, center lines indicate median values and whiskers represent minimum and maximum values. (**G**) Tumor Nanostring analysis of NK cell activating and inhibitory receptor genes. (**H**) Downstream pathway analysis following dosing of anti-muPD-1-muGITR-L bispecific in CT26 tumor syngeneic model. Gene ontology biological processes (GO-BP) enrichment analysis of TILs between anti-PD-1-GITR-L and combo-treated mice shows enriched pathways (hypergeometric test, adjusted P-values obtained by Benjamini-Hochberg procedure). Tumors were collected 24 hrs following second dose. Source data

**Extended Data Fig. 8:**
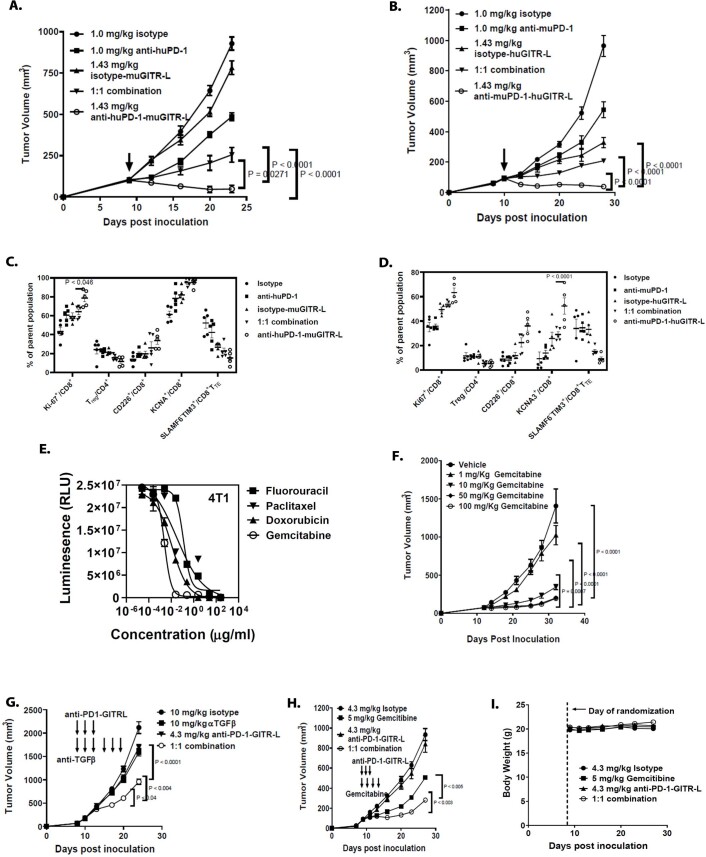
The anti-huPD-1-muGITR-L and anti-muPD-1-huGITR-L bispecifics have different bioactivity in vivo in comparison to anti-PD-1 plus isotype-GITR-L combination and monotherapies in B16F10 tumor syngeneic models in huPD-1 and huGITR HO Tg mice, and bioactivity, anti-tumor efficacy and toxicity of gemcitabine and TGFβ blockade in combination with anti-PD-1-GITR-L in 4T1 mouse syngeneic tumor model. (**A** and **B**) Growth inhibition of B16F10 in huPD-1 HO Tg mice (**A**) and in huGITR HO Tg mice (**B**) following indicated treatments and doses (i.p. frequency indicated by arrows). Each point on the curve represents the mean tumor volume for each group (n = 8 mice). (**C** and **D**) Flow cytometry analysis of tumor infiltrated T cells collected from both models (120 hours post dose, n = 5 mice). Ki67^+^, CD226^+^, KCNA3^+^ and SLAMF6^-^TIM3^+^ are shown as a percent of CD8^+^ T cells ((**C**) huPD-1 HO Tg, and (**D**) huGITR HO Tg). Data are presented as mean values +/- SEM. Statistical significance was calculated with two-way ANOVA with Tukey’s correction for multiple comparisons (statistics refer to anti-PD-1-GITR-L vs combo). (**E**) *In vitro* cell killing of 4T1 mouse syngeneic cell line to selected chemotherapeutic agents (fluorouracil, paclitaxel, doxorubicin and gemcitabine) using CellTiter-Glo cell viability assay (n = 3 technical cell culture replicates within a single experiment). (**F**) Dose range titration of gemcitabine in 4T1 model at indicated doses (i.p. Q3DX4). (**G** and **H**) Growth inhibition of 4T1 cells in syngeneic mice by combination of anti-muPD-muGITR-L with anti-TGFβ (**G**) or gemcitabine (**H**) following indicated treatments and doses (i.p., frequency indicated by arrows). Data are presented as mean values +/- SEM. (**I**) Change of mice body weight following indicated treatments. Gemcitabine was dose i.p. Q3DX4 and anti-muPD-1-muGITR-L bispecific was dose i.p. 3qW for 1 week. Each point on the curve represents the mean tumor volume for each group (n = 10 mice). Statistical significance was calculated with two-way ANOVA with Tukey’s correction for multiple comparisons [statistic refer to gemcitabine (100 mg/kg) vs vehicle, anti-PD-1-GITR-L+ TGFβ vs anti-PD-1-GITR-L; and anti-PD-1-GITR-L vs gemcitabine]. Source data

Tumor growth inhibition induced by anti-muPD-1-muGITR-L was different from the effect of single agents (anti-muPD-1 and isotype-muGITR-L, where isotype variable domain is an anti-huCMV, clone MSL109) and a 1:1 combination in anti-PD-1-resistant syngeneic tumor models including CT26, EMT6 and JC (Fig. 6a–c). Anti-PD-1–GITR-L-treatment also showed prolonged overall survival for CT26, EMT6 and JC tumor-bearing mice (~70% of mice were tumor free for >50 days; Extended Data Fig. 6h–j). The bispecific also showed enhanced bioactivity in comparison to the combination in the JC model, where either the isotype-muGITR-L or the anti-GITR antibody has effector functions (mIgG2a) (Extended Data Fig. 7a). An increase in the percentage of ICOS^+^ and Ki67^+^ in CD8^+^ T cells isolated from TDLNs correlates well with the antitumor efficacy of anti-muPD-1-muGITR-L in the CT26 and JC models (Fig. 6d,e). In addition to enhanced T cell activation and proliferation, the bispecific induced an antigen-specific memory T cell response, as shown by rejection of multiple tumor rechallenge inoculations in anti-PD-1–GITR-L-treated CT26 regressors (Fig. 6f). No full tumor regressions were observed following treatment of mice with a combination. Accumulation of antigen-specific T cells has also been observed with peptide–major histocompatibility complex tetramer staining (Fig. 6g). An increase in both the number of CT26-specific GZMB^+^ lymphocytes and percentage of CT26 cell killing has been observed in CD8^+^ T cells isolated from TDLNs of anti-muPD-1-muGITR-L-treated mice in comparison to the combination (Fig. 6h,i). Also, when tumors were assessed by IHC, GZMB^+^ cell numbers were higher in the anti-muPD-1-muGITR-L-treated group compared to either the combination- or isotype control-treated groups (Fig. 6j). In addition, anti-muPD-1-muGITR-L increased GZMB production at a higher percentage by CD8^+^ T than NK cells, which suggests that the bispecific mainly engages CD8^+^ T cells as part of their MoA (Fig. 6k). The dependence of CD8^+^ T cells for the MoA of anti-muPD-1-muGITR-L was confirmed by the absence of efficacy in the EMT6 model following CD8^+^ T cell depletion studies. In comparison, CD4^+^ T cells are apparently dispensable for the MoA of the bispecific while they play a role in that of the combination (Fig. 6l). The anti-muPD-1-muGITR-L induced a higher propensity for immune activity within tumors than both the combination and monotherapies (defined as genes with *P* < 0.05 by Student’s *t-*test) assessed by NanoString gene expression analysis following treatment in the CT26 model (Extended Data Fig. 7b). Compared to the combination treatment, anti-muPD-1-muGITR-L resulted in differential expression of 335 of 751 genes evaluated (Extended Data Fig. 7c). Immune cell gene quantification analysis revealed upregulation of gene signatures associated with adaptive (CD8^+^ T cell) and innate (NK cell) immune response, and cytotoxicity following treatment with anti-muPD-1-muGITR-L (Extended Data Fig. 7d–f). Analysis of individual genes revealed that anti-muPD-1-muGITR-L treatment increased the expression of *CD8a* and *GZMB*, which are critical signs/mediators of CD8^+^ T cell infiltration and cytotoxicity relative to both monotherapies and combinations (Fig. 6m,n). Also, different genes related to activating (*KRLK1*, *KLRC2*, *NCR1*) and inhibitory (*KLRD1*, *KLRA2*, *KLRC1*, *KLRA7*, *KLRG1*) receptor pathways on NK cells (Extended Data Fig. 7g) have been observed only for the bispecific. A Gene Ontology biological processes enrichment analysis indicated upregulation of genes involved in the cellular response to IFN-γ and MAP kinases (ERK1/ERK2), chemokine-mediated signaling pathways, lymphocyte chemotaxis and immune/inflammatory responses (Extended Data Fig. 7h). Moreover, chimeric bispecifics have shown different bioactivity in vivo than both the combination and monotherapies in the B16F10 tumor syngeneic model in huPD-1 and huGITR homozygous transgenic mice (Extended Data Fig. 8a–d). These results suggest that anti-muPD-1-muGITR-L resulted in a higher propensity of immune activity compared to combination therapy. Also, enhanced antitumor activity was observed with the combination of anti-muPD-1-muGITR-L with anti-TGFβ and gemcitabine in an immune checkpoint blockade (ICB)-resistant model (4T1), as previously shown with other immunotherapy agents (Extended Data Fig. 8e–i)^37,38^.

**Fig. 7:**
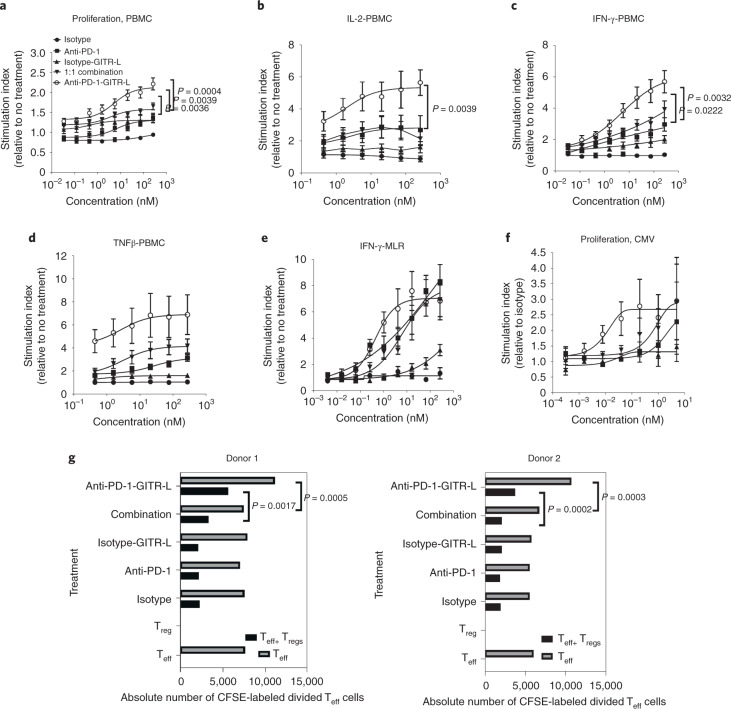
The anti-huPD-1-huGITR-L bispecific enhances in vitro PBMC costimulation and reverses T_reg_ suppressive activity in comparison to the combination of anti-PD-1 plus GITR-L. **a**–**d**, Human PBMC costimulation assay following the indicated treatments (in the presence of anti-CD3). Cells and supernatants were harvested/collected for assessment of proliferation (**a**) (*t* = 48 h, half-maximal effective concentration (EC_50_) = 3.3 nM, *n* = 12 donors) and IL-2 (**b**) (*t* = 48 h, EC_50_ = 1.5 nM, *n* = 8 donors), IFN-γ (**c**) and TNF-β (**d**) (*t* = 96 h, EC_50_ = 3.9 nM and EC_50_ = 2.6 nM, and *n* = 12 and 8, respectively). Data presented as mean ± s.e.m. (*n* = 2 technical cell culture replicates within a single experiment). **e**,**f**, IFN-γ secretion (**e**) (*t* = 120 h) in autologous CD4^+^ T cell MLR (*n* = 7 donors, EC_50_ = 0.45 nM) and cell proliferation (**f**) (*t* = 72 h, EC_50_ = 0.8 nM) in CMV antigen recall assay (*n* = 3). Data presented as mean ± s.e.m. (*n* = 2 technical cell culture replicates within a single experiment). **g**, T_reg_ suppression assay (*n* = 2 donors) measuring absolute number of CFSE-labeled divided T_eff_ cells in response to anti-CD3 and indicated treatments in the presence and absence of T_reg_ cells (*t* = 72 h, T_eff_/T_reg_ = 1). Statistical significance was calculated by two-way ANOVA with Tukey’s correction for multiple comparisons (statistics refer to anti-PD-1–GITR-L bispecific versus combination; representative data of *n* = 2 independent experiments with similar results). Source data

In human PBMC costimulation assays, treatment with anti-huPD-1-huGITR-L resulted in enhanced dose-dependent proliferation, IFN-γ, IL-2 and TNF-β in comparison to single and 1:1 combination treatments (Fig. 7a–d). Also, the bispecific induced an increase in IFN-γ production in autologous CD4^+^ T cell mixed-lymphocyte reactions (MLR) and T cell proliferation in human PBMCs in an antigen recall response to cytomegalovirus (CMVpp65) (Fig. 7e,f). The effect of anti-huPD-1-huGITR-L on T_reg_ cell activity was assessed in suppression assays, demonstrating that treatment with the bispecific resulted in a higher percentage of restoration of T_eff_ (CD4^+^CD25^–^) cell proliferation in comparison to single and combination treatments (Fig. 7g). Also, T_eff_ cell proliferation in the absence of T_reg_ cells has been observed only following treatment with anti-huPD-1-huGITR-L, due to optimal GITR crosslinking. Anti-PD-1 treatment did not show any enhanced bioactivity resulting from the absence of PD-L1^+^ cells in this assay setup, while GITR-L agonism was also limited due to suboptimal GITR crosslinking. These results suggest that anti-huPD-1-huGITR-L induces a combination of T_eff_ resistance and/or inhibition of T_reg_ suppressive activity.

### Anti-PD-1–GITR-L induces proliferation of CD4^+^ T cells in NHP

**Fig. 8:**
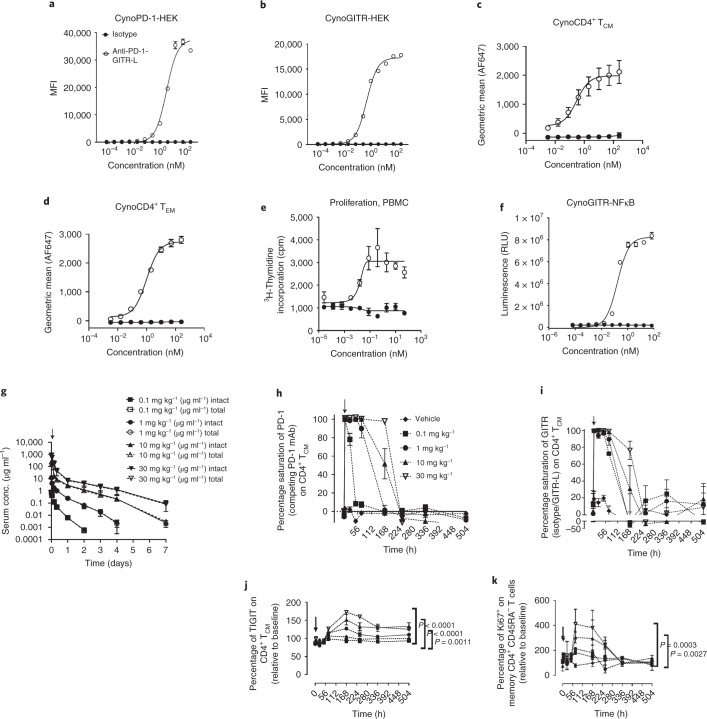
In vitro and in vivo crossreactivity of anti-huPD-1-huGITR-L to cynomolgus monkeys. **a**,**b**, Binding of anti-huPD-1-huGITR-L to cynomolgus PD-1 (**a**) and GITR (**b**) transfected HEK293S cells (*n* = 3 technical cell culture replicates within a single experiment). **c**,**d**, Binding of anti-huPD-1-huGITR-L to cynomolgus CD28^+^CD95^+^ central memory (**c**) and CD28^–^CD95^+^ effector memory CD4^+^ T cells (**d**) (*n* = 3 donors). **e**, Cynomolgus monkey PBMC proliferation assay (*n* = 3 donors) following the indicated treatments with threefold titration (in the presence of anti-CD3; *t* = 48 h). **f**, NFκB signaling in HEK293-NFκB-cynoGITR^+^ reporter assay following threefold titration with the indicated treatments (*n* = 3 technical cell culture replicates within a single experiment). **g**–**k**, Serum concentration versus time profile (**g**), saturation of PD-1 (**h**) and GITR (**I**) and upregulation of TIGIT (**j**) and Ki67 (**k**) in CD4^+^ memory T cells following IV bolus administration of anti-huPD-1-huGITR-L bispecific at indicated doses in cynomolgus monkeys (*n* = 3 NHP). Data presented as mean ± s.e.m. Statistical significance was calculated by two-way ANOVA with Tukey’s correction for multiple comparisons (statistics refer to anti-PD-1–GITR-L bispecific (30 mg kg^–1^) versus vehicle). Source data

**Extended Data Fig. 9:**
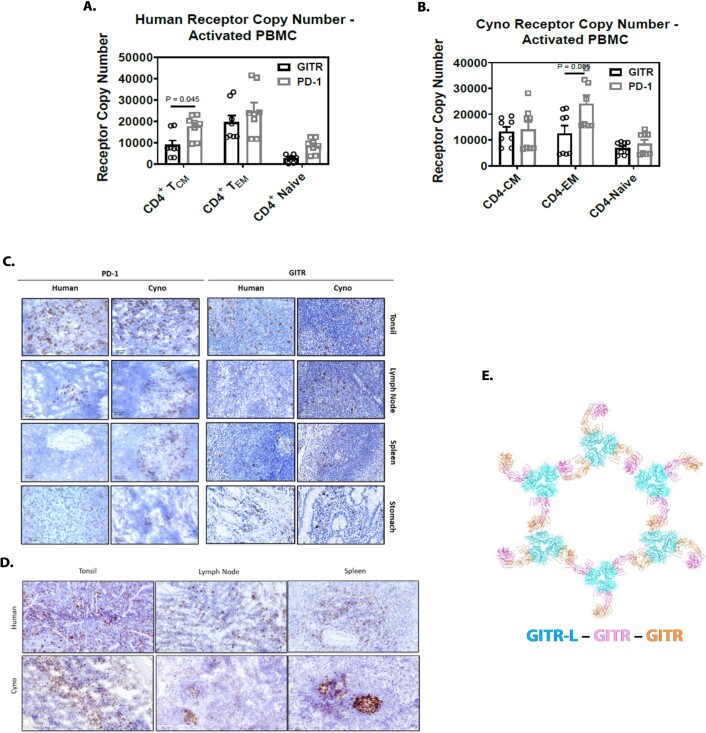
PD-1 and GITR receptor copy number, expression and anti-PD-1-GITR-L cross-reactivity on normal human and cynomolgus monkey tissues by IHC, and Hexagonal conformation model of GITR-L-GITR complex. (**A** and **B**) GITR and PD-1 receptor copy number on PHA activated (t = 48 hrs) human (A) and cynomolgus monkey (B) PBMCs (n = 8 donors per group). Data are presented as mean values +/- SEM. Statistical significance was calculated with two-way ANOVA with Tukey’s correction for multiple comparisons (statistic refer to GITR vs PD-1 expression). (**C**) Representative human and Cyno PD-1 and GITR expression by IHC (representative staining of n = 2 tissues) on tonsil, lymph node, spleen, and stomach tissues. FFPE tissue samples were used for PD-1 and GITR (Human TMA: ZPL2 and ZPL3, and Cyno: I09447 and I08762) staining. Scale bars indicate 50 µm (PD-1) and 60 µm (GITR) (original magnification 40x). (**D**) Representative anti-huPD-1-huGITR-L bispecific binding on normal human and cynomolgus monkey tissues using human to human pre-complex conjugated IHC staining method. Scale bars indicate 200 µm (original magnification 20x, representative staining of n = 2 tissues). (**E**) GITR-L trimeric units are shown in light blue, and GITR monomeric units from different GITR trimers are shown in pink and orange. Source data

To establish in vivo proof of mechanism and to identify PD biomarkers in nonhuman primates (NHP), the cynomolgus monkey crossreactivity of anti-huPD-1-huGITR-L was investigated. A high sequence identity was observed between human and cynomolgus macaque PD-1 and GITR (Supplementary Table 8). Also, similar PD-1 and GITR receptor numbers, expression levels and anti-PD-1–GITR-L tissue crossreactivity were found in human- and cynomolgus monkey-activated T cells in PBMCs and tissues (Extended Data Fig. 9a–d and Supplementary Table 9). The anti-huPD-1-huGITR-L binds to cell-surface cynomolgus PD-1 and GITR-transfected cells, to activated cyno PBMCs and to recombinant protein antigen (Fig. 8a–d and Supplementary Tables 10 and 11). Treatment of cynomolgus monkey PBMCs with anti-huPD-1-huGITR-L induced proliferation and NFκB signaling in a HEK293 cynoGITR^+^ reporter cell line (Fig. 8e,f). In conclusion, anti-huPD-1-huGITR-L binds to human and cynomolgus monkey PD-1 and GITR with similar binding affinity and induces similar signaling transduction, which supports the use of cynomolgus monkeys as a relevant species for identification of PD biomarkers of anti-huPD-1-huGITR-L.

The PK of human anti-huPD-1-huGITR-L was evaluated in a single-dose, non-good laboratory practices PK/PD study in naïve cynomolgus monkeys. In that study, three monkeys in each group received a single intravenous (IV) bolus dose. Similar serum concentrations of anti-huPD-1-huGITR-L were determined by intact and total analytical assays. A noncompartmental analysis showed a nonlinear PK profile of 0.1–1.0 mg kg^–1^ and a linear PK profile of 10–30 mg kg^–1^, with a half-life ranging from 5 to 18 h. A decrease in clearance with increasing doses was observed, ranging from 42 to 12.1 ml h^–1^ kg^–1^. Both *C*_max_ and AUC_inf_ increased in a dose-proportional manner, from 1 to 30 mg kg^–1^ (Fig. 8g and Supplementary Table 12). Fluorescent activated cell sorter (FACS)-based PD assessments of target engagement and downstream immune changes were evaluated. Complete saturation of PD-1 and GITR on CD4^+^ T_cm_ cells with anti-huPD-1-huGITR-L was observed 4 h post dose, followed by dose-dependent desaturation at 168 h (Fig. 8h,i). The anti-huPD-1-huGITR-L induced a dose-dependent TIGIT increase in CD4^+^ T_cm_ cells sustained at both 10 and 30 mg kg^–1^ (Fig. 8j). Furthermore, a maximal and dose-dependent Ki67 increase was observed at day 7 post dose in CD4^+^CD45RA^–^ memory T cells at 1.0, 10 and 30 mg kg^–1^ (Fig. 8k). Pharmacodynamic markers observed in naïve cynos represent a low subclinical immunological activity that had been enhanced by the bispecific. Minimal changes in plasma cytokines and chemokines were observed (<50 pg ml^–1^) between placebo- and anti-huPD-1-huGITR-L-treated groups, demonstrating the absence of toxicity. Exposure of anti-huPD-1-huGITR-L in the cynomolgus monkey results in a consistent relationship between target saturation and modulation of immunologically relevant PD effects, demonstrating conserved PD action between the surrogate anti-muPD-1-muGITR-L in mice and anti-huPD-1-huGITR-L in NHP that validates the MoA.

## Discussion

Antibody-based ICB has transformed cancer therapy over the past 25 years. On the other hand, agonistic immunotherapies that boost downstream T cell signaling have proven much harder to develop despite great promise. Specifically, agonistic antibodies against costimulatory receptors have shown limited therapeutic effect in several early-stage clinical trials^39^. Lack of optimal TNFR clustering and signaling with FcγR-binding-dependent antibody-based therapeutics may explain their limited activity in humans. Thus, because many patients do not benefit from ICB, alternative TNFR agonistic therapies are still being sought for development.

The GITR signaling pathway is an attractive immuno-oncology target, due to its capacity to promote effector T cell functions and regulatory T cell suppression. Optimal GITR oligomerization mediated by an engineered dodecavalent GITR-L hexameric Fc construct has been shown critical in the induction of T cell activation, probably by forming a hexagonal GITR-L–GITR complex arrangement. As shown in the X-ray structure, a noncovalent GITR dimer interaction may mediate a large network of adjacent GITR-L–GITR complexes where distances between them may dictate the intracellular distances between TRAF RING domains and the signalosome for transduction of signals into T cells. Although we did not observe a hexagonal arrangement in the crystal packing of the GITR-L–GITR complex structure, a model can approximate a hexagonal network (Extended Data Fig. 9e) as has been shown for other TNFR complexes^40,41^. These results suggest that GITR clustering is critical and sufficient to induce optimal signal transduction. Unlike conventional GITR antibodies, which are FcγR binding dependent for crosslinking/agonism and ADCC, our bispecific induced PD-1-dependent GITR clustering and signaling in primed, antigen-specific double-positive T cells.

The surrogate anti-muPD-1-muGITR-L with null effector function demonstrated dose-dependent antitumor efficacy in CT26 and EMT6 tumor models following a single dose. A half-life of approximately 1 day and partial target saturation in the circulation was sufficient to drive an extended PD effect in vivo, inducing a dose-dependent, immunologically T cell-driven mechanism after a single dose of the bispecific. TIGIT upregulation has been considered a marker of T cell activation, because an increase in TIGIT expression has been observed previously following treatment with anti-CD3/CD28 (ref. ^42^). An increase in the frequency of ICOS^+^CD4^+^ T cells is considered a PD biomarker of anti-CTLA-4 and PEG-IL-2 (NKTR-214)^43,44^, while an increase in the percentage of Ki67^+^CD8^+^ T cells predicted durable clinical response following anti-PD-1 therapy^45,46^. Anti-PD-1–GITR-L dosing in mice decreased intratumoral T_regs_, which has been also observed following anti-GITR treatment in patients, potentially explained by an increase in plasticity and conversion to an inflammatory effector T cell phenotype^9,47–49^. A single-cell mRNA-seq analysis revealed that anti-PD-1–GITR-L reduces exhausted TOX^+^CD8^+^ T cells within the TME, which agrees with the reduction in intratumoral SLAMF6^–^TIM3^+^CD8^+^ and TOX^+^CD8^+^ T_TE_ cells. High concentrations of K^+^ within tumor necrotic areas inhibit Akt/mTOR TCR signaling and T cell effector functions^50^. Upregulation of K^+^ channels on intratumoral CD8^+^ T cells following treatment with anti-muPD-1-muGITR-L may restore the ionic balance and membrane polarization on T cells by lowering K^+^ concentration and increasing Ca^2+^ uptake, leading to enhanced effector function in TILs and improved antitumor responses in mice. In vivo efficacy and PD biomarkers induced by anti-PD-1–GITR-L in mice were validated with the human bispecific in humanized single-target KI and NSG allogeneic PBMC-engrafted mouse tumor models, which supports translation of the MoA.

However, several cancer indications are considered excluded due to the low degree of lymphocyte infiltration within the TME. The anti-PD-1–GITR-L shows limited efficacy as a monotherapy in immune-excluded tumors (4T1), but we showed enhanced activity in combination with TGF-β inhibition and gemcitabine. Neutralization of TGF-β signaling complements bispecific bioactivity because it may enhance its inhibitory T_reg_ suppressive activity. Gemcitabine-mediated tumor cell killing may not only help to induce CD8^+^ T cell crosspriming but also to abrogate the activity of myeloid-derived suppressor cells and/or tumor-associated macrophages. These results support the concept that the anti-PD-1–GITR-L bispecific can potentially synergize with other immunotherapies (that is, anti-TIGIT antibodies, CAR T cells, immunocytokines and so on) in immunogenically cold cancer indications.

The anti-PD-1–GITR-L bispecific has a different MoA in comparison to the effect of monotherapies and concurrent combination in mouse models, and in in vitro human PBMC costimulation assays. The bispecific induced not only target expression crossregulation but also anti-PD-1-mediated GITR clustering and sustained T cell activation. Coengagement of PD-1 and GITR has been also demonstrated to be critical for the activity of the bispecific, based on the absence of efficacy in WT mice following treatment with the chimeric bispecifics. The anti-PD-1–GITR-L showed enhanced antitumor efficacy in comparison to anti-PD-1, even though it can induce only partial PD-1 saturation, perhaps owing to induction of GITR-mediated optimal T cell costimulation during priming. Suboptimal bioactivity has been observed with the isotype GITR-L due to the absence of optimized GITR clustering and T cell activation, as shown in our in vitro assays with the divalent Fc-[(GITR-L)_3_]_2_. The anti-PD-1–GITR-L induced a higher propensity for immune activity within TDLNs and tumors than did the combination. In addition, an antigen-specific memory T cell response was demonstrated only in bispecific-treated CT26 mice, because no full tumor full regression was observed with the combination. Our anti-PD-1–GITR-L is highly efficacious in different anti-PD-1-resistant syngeneic models and has a distinct MoA in comparison to the combination of anti-PD-1 and anti-GITR antibodies (efficacious only in the highly immunogenic, anti-PD-1-responsive MC-38), where anti-GITR (rIgG2b, FcγR effector active) mainly works by induction of T_reg_ depletion and anti-PD-1 induces PD-L1 inhibition due to prolonged saturation of PD-1 (refs. ^51,52^). The anti-PD-1–GITR-L is a PD-1-directed GITR-L that may enhance binding of GITR-L to PD-1^+^CD8^+^ T cells due to higher levels of PD-1 expression in CD8^+^ versus CD4^+^ T cells. This hypothesis has been confirmed by the upregulation of proliferation, activation and memory T cell markers, not only in CD4^+^ but also in CD8^+^ T cells, where GITR has lower expression. Also, it has been confirmed that the bispecific is fully active in the EMT6 model in the absence of CD4^+^ T cells, as previously suggested by enhanced antitumor efficacy following dosing of a pentamerized GITR-L construct in the absence of CD4^+^ T cells^53^. This confirms that only CD8^+^ T cells play a critical role regarding the MoA of the bispecific by targeting GITR agonism to PD-1^+^CD8^+^ T cells.

**Extended Data Fig. 10:**
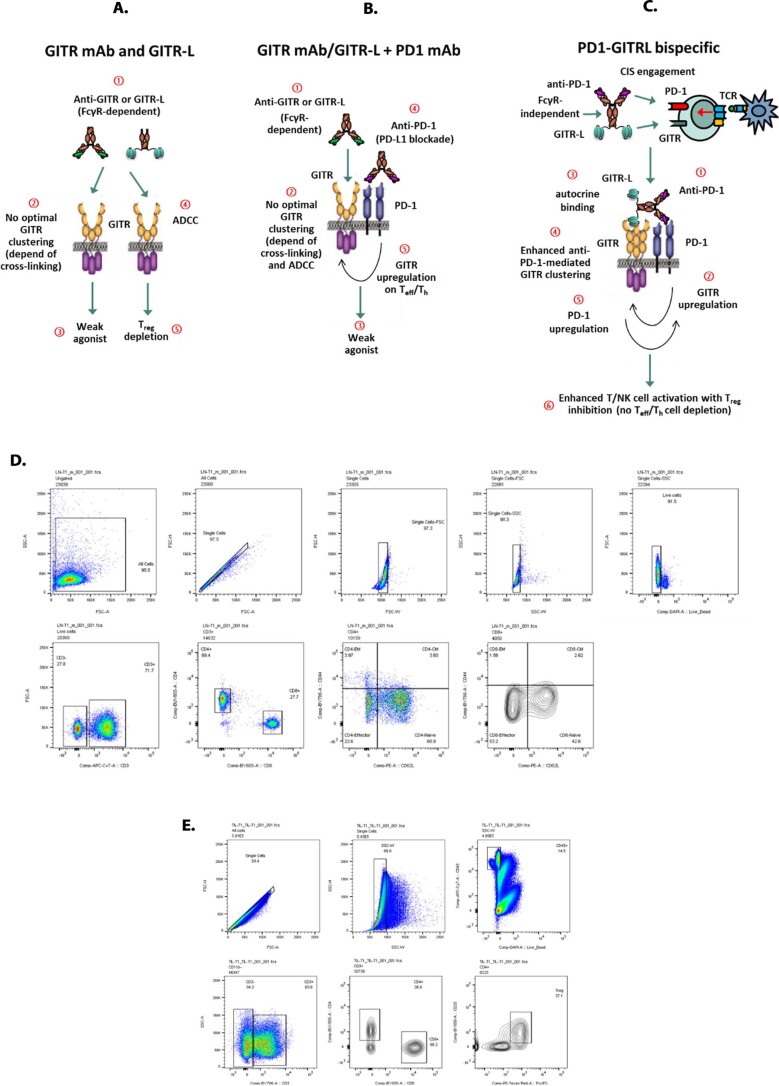
Proposed mechanism of action of FcγR-binding independent anti-PD-1-GITR-L bispecific, and Flow cytometry gating strategy for lymphocytes isolated from TLNs and TILs from CT26 and EMT6 treated mice. (**A**) Proposed MoA of FcγR-dependent anti-GITR mAb or GITR-L construct: weak clustering/agonism. (**B**) Proposed MoA of the combination of FcγR-dependent anti-GITR mAb or GITR-L construct plus αPD-1 mAb: weak clustering/agonism even upregulation of GITR after αPD-1 engagement. (**C**) Anti-PD-1-GITR-L bispecific works by inducing a FcγR-binding independent anti-PD-1-mediated GITR clustering/agonism (interaction in *trans* has not been represented in diagram). (**D** to **E**) Flow cytometry gating strategy for lymphocytes isolated from TLNs (**D**) and TILs (**E**).

This ‘activity-by-targeting’ concept has previously been described^54^. A proposed MoA of anti-PD-1–GITR-L is shown in Extended Data Fig. 10a–c.

Because anti-huPD-1-huGITR-L has similar affinity to, and IHC crossreactivity and bioactivity on, lymphocytes from both human and NHP, the cynomolgus monkey was chosen to identify in vivo PD biomarkers. Overall, the data generated in cynomolgus monkeys are indicative of anti-huPD-1-huGITR-L being pharmacodynamically active in this species, which supports the conclusion that T cell activation and proliferation observed in mice and humanized models translates to NHP.

Overall, our data support the conclusion that bispecific agonists engineered to induce optimal TNFR clustering independent of FcγR binding are more potent than monoclonal antibodies and TNFR ligands. Furthermore, our comprehensive in vitro and in vivo data, along with our PD biomarkers, all support the conclusion that the biology observed in mice translates to NHP and humanized systems. In conclusion, the anti-huPD-1-huGITR-L bispecific represents a promising immunotherapeutic approach to overcoming immune escape in PD-(L)1-refractory patients, by optimization of clustering-mediated costimulation of antigen-specific T cells.

## Methods

The research presented in this report complies with all relevant ethical regulations. All animal procedures were performed in accordance with protocols approved by the Global Animal Welfare internal Institutional Animal Care and Use Committee, and were performed in accordance with guidelines in the Guide for the Care and Use of Laboratory Animals (National Resource Council, 2018).

### Transfected and mouse cancer cell lines

Human and cyno PD-1- and GITR-expressing HEK cells were generated by transfection of human and cyno PD-1 and GITR expression vectors. The human PD-1 (huPD-1)-expressing nuclear factor of activated T cells (NFAT) reporter Jurkat cell line (catalog no. CS187102, Promega) and human PD-L1-expressing CHOK1 activator cells (catalog no. CS187108, Promega) were obtained from commercial sources. HEK 293-transduced NFκB reporter cell lines expressing human and cynomolgus GITR proteins were internally generated. The adherent colorectal carcinoma cell line CT26 (catalog no. CRL-2638, ATCC), mammary gland adenocarcinoma cell line JC (catalog no. CRL-2116, ATCC), melanoma carcinoma cell line B16F10 (catalog no. CRL-6475, ATCC) and adherent mammary carcinoma cell line EMT6 (catalog no. CRL-2755, ATCC) were obtained from commercial sources. The adherent colorectal carcinoma cell line MC-38 was obtained from the University of Chicago (no. L-085-2016/0, NIH). The adherent human prostate cancer cell line PC-3 (catalog no. CRL-1435, ATCC) and the colorectal carcinoma cell line HCT-116 (catalog no. CCL-247, ATCC) were also obtained from commercial sources.

### GITR–GITR-L complex structure determination

DNA encoding the full-length GITR and GITR-L extracellular domains was cloned into separate modified mammalian pHybE expression vectors. Coexpression and cotransfection were carried out by transient transfection of HEK 293-EBNA cells using a 3:1 ratio of GITR-L to GITR vector, and a 4:1 ratio of polyethylenimine (catalog no. 23966-1, Polysciences) to DNA. Protein complexes were captured by immobilized metal affinity chromatography, and were further purified by size-exclusion chromatography using a Superdex 200. The GITR–GITR-L complex was crystallized using the sitting-drop vapor-diffusion technique in 96-well MCR two-well plates (Hampton Research). Crystals were grown in 20% (w/v) polyethylene glycol 1500 and 0.1 M citric acid pH 3.5. Diffraction data were collected under gaseous nitrogen at 100 K at the Advanced Photon Source Beamline 17-ID. Diffraction intensities were processed using autoPROC, and the structure was solved by sequential molecular replacement with coordinates from Protein Data Bank (PDB) code 2Q1M and 3WVT using Phaser within the CCP4 program suite. The model was rebuilt using COOT and refined against structure factors using the programs REFMAC5 and autoBUSTER. Figures were prepared using the program PyMOL (Schroedinger, LLC). Atomic coordinates for the complex have been deposited in PDB with accession code 7LAW. Diffraction and refinement statistics are listed in Supplementary Table 13.

### Negative-stain TEM

Dried grids were imaged on a JEOL 1400 TEM operating at 120 keV using an UltraScan4000 CCD camera at a nominal magnification of ×30,000 and pixel size of 3.71 Å at the specimen level. In total, 112 micrographs were collected using Leginon^55^ at a defocus range of 0.5–2.0 µm. Micrographs were processed and class averages generated using Xmipp^56^ from the Scipion software package^57^. A total of 26,770 particles were selected automatically, followed by several rounds of two-dimensional (2D) classification to select the best particles. A final set of 20,657 particles was accepted and grouped into 51 classes.

### Binding affinity assays

Human PD-1-, human GITR-HEK293- and human FcγRI-, FcγRIIA-, FcγRIIB-, FcγRIII-F158- and V158-expressing CHOK1 cells were incubated with a serial dilution of antibodies. Cells were resuspended in secondary R-Phycoerythrin AffiniPure F(ab’)_2_ Fragment Goat Anti-Human IgG, Fcγ specific and Fab specific. C1q binding was determined by ELISA. Costar high-binding plates were coated with serial dilutions of samples and incubated with 2 µg ml^–1^ human complement protein C1q (catalog no. A400, Quidel). Plates were incubated with horseradish peroxidase (HRP)-sheep anti-human C1q (catalog no. ab46191, Abcam) and developed by the ddition of TMB substrate (catalog no. TMBW-1000-01, SurModics). Absorbance at 650 nm was measured using a VERSAmax reader (Molecular Devices).

Human PBMCs were isolated from buffy coats using Ficoll and treated with CD3/CD28 beads. For flow cytometry, a combination of fluorescent-labeled Abs was used. The geometric mean of anti-PD-1–GITR-L^+^, anti-PD-1^+^ and isotype huGITR-L^+^ on immune cell subsets was determined using either Alexa Fluor 647-labeled molecules or isotype control. Cells were acquired on a LSR Fortessa (BD) and analyzed using FlowJO software. Cell frequency was analyzed on subsets of CD4^+^ T cells: CD45RA^–^/CCR7^+^ central memory T cells and CD45RA^–^/CCR7^–^ effector memory T cells. The binding kinetics for recombinant soluble human PD-1, GITR and FcRn/β2m were determined by surface plasmon resonance (SPR)-based measurements recorded on a Biacore T200 (GE Healthcare) using an anti-human heavy and light (H+L) chain capture antibody. For FcRn/β2m, samples were directly immobilized by amine coupling. Recombinant extracellular domains of human PD-1 and GITR were purchased from Creative Biomart and further purified by gel filtration. The human FcRn/β2m heterodimer was internally generated. FcRn/β2m binding measurement was conducted in a running buffer adjusted to pH 6.0. For anti-human H+L capture chip preparation, approximately 2,000 RU of goat anti-human H+L polyclonal antibody was directly immobilized across a CM5 biosensor chip using a standard amine coupling kit. Data were processed and fitted globally to a 1:1 binding model using Biacore T200 evaluation software to determine the binding kinetic rate constants *k*_a_ (1/Ms) and *k*_d_ (1/s), and the equilibrium dissociation constant *K*_d_ (M).

For the cell bridging assay, huGITR-HEK293 cells were labeled with 5 µM CellTrace Violet Dye per 10 × 10^6^ cells ml^–1^, while huPD-1-HEK293 cells were labeled with 0.25 uM CellTrace CSFE per 10 × 10^6^ cells ml^–1^. After labeling, 1 × 10^5^ fluorescent-labeled cells were incubated with 2.5 µg ml^–1^ treatment for 30 min. Cells were acquired and analyzed as previously described.

### NFκB/ NFAT reporter assays

For the NFκB reporter assay, 100,000 cells were seeded in a 96-well flat-bottom plate and serial dilution was performed for antibodies. After 24 h, luciferase activity was quantified using BriteLite Plus (catalog no. 6066761, PerkinElmer) and measured using an EnSpire Alpha multimode plate reader (catalog no. 2300, PerkinElmer). Jurkat-NFκB-GITR^+^ stable cells were generated by transduction using lentivirus particles. Transduced cells were sorted and screened for human GITR and NFκB expression, then incubated with anti-CD3 for 48 h to induce upregulation of PD-1 expression, which was later confirmed by flow cytometry. Next, a serial dilution of anti-huPD-1-huGITR-L was added to cells and luciferase activity was evaluated using Nano-Glo Luciferase (catalog no. N1120, Promega). Cells were transferred to flat-bottom plates and substrate was added before measurement using an EnSpire Alpha multimode reader. For the NFAT reporter assay, 4 × 10^5^ PD-L1-expressing CHO K1 activator cells were plated on 96-well flat-bottom plates and incubated overnight at 37 °C with a serial dilution of antibodies and 40 µl of 1.25 × 10^6^ cells ml^–1^ human PD-1 NFAT reporter Jurkat cells. After 6 h, 80 µl of Bio-Glo reagent was added to each well and plates were incubated for 5 min at ambient temperature. Luminescence was measured as previously described.

### Binding affinity and bioactivity assays of surrogate bispecifics

Mouse PD-1- and GITR-expressing HEK 293S cells were incubated with a R-Phycoerythrin AffiniPure F(ab’)_2_ fragment goat anti-mouse IgG, Fcγ specific, to analyze binding affinity, then 2 × 10^5^ mouse splenocytes per well (Balb/c) were seeded on round-bottom plates with 0.5 µg ml^–1^ anti-mouse CD3. Splenocyte proliferation and IFN-γ release were tested with a titration of mouse bispecifics. After 72 h, supernatant was collected and analyzed using an AlphaLISA kit while cells were pulsed with 0.25 µCi ^3^H-thymidine overnight to measure the levels of T cell proliferation. A HEK 293-NFκB-muGITR^+^ reporter cell line was used to measure NFκB signaling using BriteLite and an EnSpire Alpha multimode plate reader. An IL-2 blocking reporter assay was used to evaluate PD-1–PD-L1 blocking properties.

### In vivo efficacy in mouse syngeneic tumor models

Five- to six-week-old WT Balb/c (and C57BL/6) female mice were obtained from Taconic. Tumor cells were injected SC into the right flank of Balb/c mice: either 2.5 × 10^5^ CT26 (catalog no. CRL-2638, ATCC) or 1.0 × 10^6^ EMT6 (catalog no. CRL-2755, ATCC) cells. Primary end points, tumor size and survival body weight, were measured twice weekly and mice were euthanized when tumor volume exceeded 12.5% of their body weight (~2,500 mm^3^). Maximal tumor size was not exceeded in any mouse study. Animals were examined for toxicity by clinical observations and body weight. Mice (and rats) were housed under specific-pathogen-free conditions in a facility accredited by the American Association for Accreditation of Laboratory Animal Care, International. Mice were housed in an environment with temperature ranging 68–76 °F, humidity 30–45% and a 14/10-h light/dark cycle. Mice were randomized into five treatment groups of *n* = 6 mice per group when tumors averaged 85 mm^3^ on days 11 and 7. Dose titration of anti-muPD-1-muGITR-L and controls was performed on day 11 (CT26 and JC) and day 7 (EMT6) by IV injection. Difference in molecular weight was considered for selected treatments. To evaluate the development of immunological memory, bispecific-treated CT26 mice exhibiting complete regression were rechallenged on days 56, 172 and 273 with 0.5 × 10^5^ CT26 cells. Accumulation of antigen-specific T cells was analyzed by H-2Kb MuLV gp70 tetramer-PE staining (catalog no. TB-M507-1, MBL) in fully regressed mice (at day 273). For T cell depletion studies, 200 µg of anti-CD8 (clone 2.43) and anti-CD4 (clone GK1.5) rat IgG2b mAbs was administered on days 5, 7 and 9 after establishment of EMT6 tumors. Tumor growth inhibition by a combination of anti-muPD-muGITR-L and anti-TGF-β (1D11-mIgG1) or gemcitabine was performed in the 4T1 model. Anti-TGF-β was administered three times per week for 2 weeks, gemcitabin every third day (four doses in total) and anti-muPD-1-muGITR-L three times daily for 1 week (all intraperitoneally (IP)). Measurement of tumor growth was assessed every 3–5 days by standard caliper measurement, and tumor growth volume was calculated using the formula length × width × height/2. Data calculations were made and stored using Study Log2.1.1.

### Pharmacokinetics analysis

CT26 and EMT6 tumor-bearing mice (*n* = 4) were given a single IV injection of anti-muPD-1-muGITR-L at the indicated doses. Microbleed samples were taken at 30 m, 3 h and 1, 3, 4 and 7 days. Plasma drug levels were determined by ELISA. Plates were coated with mPD-1-Fc (1 µg ml^–1^, overnight, R&D systems) and a biotinylated anti-muGITR-L antibody (BioLegend) with SA-HRP. Anti-muGITR-L measures intact levels of anti-PD-1–GITR-L in plasma (lower level of quantitation, 0.1–100 ng ml^–1^). Concentration–time data were analyzed using noncompartmental methods, and PK parameters were estimated or calculated using WinNonlin Model 201 (WinNonlin, v.5.2.1, Pharsight). Values of *R*^2^ or *R*^2^ adjusted ≥ 0.80 (where *R* is the correlation coefficient) were required for acceptance of Lambda *z* (*λz*) estimates in noncompartmental analyses.

### Pharmacodynamics analysis

To evaluate the PD effects of anti-muPD-1-muGITR-L in CT26, EMT6 and JC tumor-bearing mouse models (*n* = 5 per PD time point), tissues were harvested from mice treated with anti-muPD-1-muGITR-L and from controls. For blood PD, FACS Lysing Solution was diluted with reagent-grade water to 1×, and 20 µl of the antibody mix was added to each Trucount Absolute Counting tube (catalog no. 340334, BD). Next, 50 µl of well-mixed, anticoagulated whole blood was added to the side of the tube just above the retainer. CD4^+^ T cells were assessed for expression of GITR and PD-1 using Alexa Fluor 488-labeled anti-muPD-1 and isotype/GITR-L. The percentage of GITR and PD-1 saturation was calculated by dividing the percentage of GITR or PD-1 after dosing by the percentage in isotype-treated mice at the same post-dosing times. Absolute cell counts were determined using this equation: ((no. of events in region containing cell)/(no. of events in absolute count bead region)) × ((no. of beads per test)/(test volume)). TDLNs were processed individually by gentle maceration between two frosted microslides and pipetting up and down to release cells thoroughly into the medium. Cells were then strained through a 70-µM pipet tip strainer into a 5-ml polystyrene round-bottom tube, and centrifuged at 1,200 r.p.m. for 5 min. The flow cytometry panel includes markers for CD45 (clone 30-F11), CD4 (clone RM4.5), CD8 (clone 53-6.7), CD62L (clone MEL-14), ICOS (clone 7E.17G9), CD44 (clone IM7), TIGIT (clone 1G9) and Ki67 (clone 16A8). The flow cytometry gating strategy is described in Extended Data Fig. 10d. CT26-specific, GZMB-positive cells were quantified in CD8^+^ T cells isolated from TDLNs using the mouse GZMB ELISPOT kit (catalog no. XEL1865, R&D). CD8^+^ T cells isolated from TDLNs were plated in ELISPOT plates at 2 × 10^5^ cells per well. After 24 h in incubation, ELISPOT plates were processed using a biotinylated anti-GZMB Ab as a secondary antibody, streptavidin-HRP (dilution 1:100) and tetramethylbenzidine (peroxidase substrate for assay development). All assay plates were scanned and analyzed using the same preoptimized counting parameters on an S6Macro696 Analyzer with ImmunoSpot v.5.1. To quantify the target cell-killing activities mediated by tumor-specific CD8^+^ T lymphocytes, we used the flow cytometry-based CTL assay (catalog no. C10427, ThermoFisher) to detect the specific cleaved caspase-3/7 in target cells. GZMB-positive CD3^–^CD49b^+^ NK and CD8^+^ T cells isolated from tumors (in the presence of brefeldin A) were also quantified by intracellular flow cytometry. Cells were fixed and permeabilized, followed by intracellular staining with GZMB (clone GB11).

Approximately 2–10 million cells were stained from each tumor for flow cytometry with a mouse FcγR blocking reagent. Phenotyping of TiLs was performed in two staining panels, including antibiotics against the following. Panel 1: CD45 (clone 30-F11), CD4 (clone RM4.5), CD8 (clone 53-6.7) and CD62L (clone MEL-14); panel 2: CD45 (clone 30-F11), CD4 (clone RM4-5), CD25 (clone PC61) and FoxP3 (clone MF23). For FoxP3, cells were surface stained, fixed and permeabilized using a staining kit. A live/dead propidium iodide was added 10 min before acquisition. Samples were acquired and analyzed on LRSFortessa using FACSDiva software. At least 5 × 10^5^ cells were acquired per sample. The flow cytometry gating strategy is described in Extended Data Fig. 10e.

### Gene expression analysis

For NanoString gene expression analysis, all mouse tumor tissues were processed as formalin-fixed, paraffin-embedded (FFPE) blocks and run on a mouse Pancancer Immune profiling panel C3400. Raw data were analyzed by NanoString nSolver 4.0. Advanced analysis used the Danaher method^58^. The score for each cell type was centered to have a mean of 0. Abundance estimates (scores) were calculated in log_2_ scale, an increase of 1 on the vertical axis corresponding to a doubling in abundance. Genes comprising a CD8^+^ T cell signature include *CD8B1* and *CD8A*; NK cells, *NCR1* and *XCL1*; and cytotoxic cells, *GZMB*, *CTSW*, *KLRK1*, *KLRD1*, *GZMA* and *PRF1*. For single-cell mRNA-seq analysis, mice were euthanized 5 days following treatment and tumors were dissected. Single-cell suspensions were generated using a tumor dissociation kit (Miltenyi Biotec), and CD45^+^ TiLs were enriched. Sequencing was performed on a NextSeq 550 (Illumina) instrument and data were processed using CellRanger pipeline (3.0.2, 10X Genomics) for demultiplexing, barcode assignment, single-cell gene counting and cluster analysis. The reference genome, mm10(GRCm38.93), was provided by 10X Genomics. Cells with >500 unique molecule identifiers were recovered, and their gene expression data were used for downstream analysis. Cluster visualization was done using Loupe Cell Browser 3.1.1. Normalization of data was performed using the ‘LogNormalize’ method in the Seurat 4.0 package in R. Cell annotation was based on the Immgen database. Cell expression profiles were correlated to the reference expression of Immgen data and based on the highest correlation coefficient with which cells were annotated to the corresponding immune cell type. Heatmaps were generated using R with the ‘ward.D2’ clustering method. Differential gene expression analysis in the CD8 T and NK populations was performed using the Wilcoxon test with a threshold of *P* = 0.001. The UCSF Immunoprofiler Initiative is an innovative research alliance where hundreds of fresh tumor samples from different indications are analyzed by, for example, flow cytometry, RNA-seq and IHC/immunofluorescence, to characterize their immune cell composition.

### Serum liver enzymes and cytokine/chemokine assessment

Blood chemistry was measured with a VetScan VS2 analyzer using Prep Profile II rotors (catalog no. 500-0026, Abaxis). Cytokine and chemokine levels were measured using a Milliplex 24-plex assay.

### Immunohistochemistry

All mouse tumor tissues were processed as FFPE blocks. Briefly, sections were dewaxed in xylenes and rehydrated. Antigen retrieval was performed using Dako target retrieval (pH 6.0) in a pressure cooker at 125 °C for 1 min, and sections were stained using a Dako Autostainer XL. Secondary antibodies used were Dako Envision-HRP. DAB was used as the chromagen, and all tissues were counterstained using Mayers hematoxylin. All slides were digitally scanned using an Aperio AT2 digital scanner. Image analysis was performed on all digitized slides using the HALOTM image analysis program from Indica Labs. The analysis module was the Area Quantification Module v.1.0.20.1; the output analysis used was percentage stain-positive tissue area. PD-1 and GITR expression in normal tissues was also evaluated by IHC. Mouse or rabbit anti-huGITR IgG2b (AGGIE.11 or D919D; catalog no. 68014S, Cell Signaling) and anti-huPD-1 IgG2b (12A11) were used with bond polymer refine and envision detection as secondary antibody (catalog no. DS9800, Leica; catalog no. K4007, DAKO). Human GITR and PD-1 expression was also evaluated in FFPE tumor microarray tissues (Conversant). For anti-PD-1–GITR-L tissue crossreactivity, the human-to-human staining protocol was used to detect anti-PD-1–GITR-L binding on normal human tissues. Frozen normal cynomolgus primate tissues were purchased from Covance. Optimal IHC staining patterns were observed with acetone-fixed frozen tissues using anti-PD-1–GITR-L. For homozygous and heterozygous transgenic mice, GITR and PD-1 expression was also evaluated by IHC on frozen spleens with the FFPE method. Rabbit anti-human GITR IgG1 (catalog no. ab223841, Abcam), rat anti-mouse GITR IgG1 (catalog no. ab210258, Abcam), rabbit anti-human PD-1 IgG1 (catalog no. ab137132, Abcam) and rat anti-mouse PD-1 IgG1 (catalog no. ab214421, Abcam) were used.

### Multiplex immunofluorescence

FFPE human HNSCC and matching lymph node metastatic tissues were stained using multiplex immunofluorescence on the Leica Bond Autostainer with the following antibodies: PD-1 (EPR4877/2) detected with Akoya Opal 780, CD8 (SP239) with Akoya Opal 570, GITR (D919D) with Akoya Opal 520 and FoxP3 (D2W8E) with Akoya Opal 620. All slides were digitally scanned using a Vectra Polaris scanner, and image analysis was performed using HALOTM (Indica Labs).

### In vivo efficacy in transgenic and humanized mouse models

Human PD-1 and GITR complementary DNAs were inserted into mouse PD-1 and GITR exon 1, respectively, with a neomycin cassette (selection marker flanked by loxP sites for further Cre-mediated excision). Generation and injection of embryonic stem cell clones into blastocysts, chimera generation and breeding, and germline transmission screening were performed by GenOway as previously described^59^. Breeding of C57BL/6 homozygous mice was performed by Charles River Laboratories. For huPD-1 and huGITR homozygous MC-38 and B16F10, tumor growth inhibition and flow cytometry analysis were performed following treatment with isotype control and chimeric bispecifics in comparison to the surrogate bispecific in WT mice. Mixed-gender transgenic mice were used for this study (*n* = 10). Flow cytometry panel 1 included CD4 (clone RM4-5), CD8 (clone 53-6.7), TIGIT (clone 1G9), CD62L (clone MEL-14), CD44 (clone 1M7), Ki67 (clone 11F6) and ICOS (clone 7E.17G9). Panel 2 included CD4 (clone RM4-5), CD8 (clone 53-6.7), CD226 (clone 10E5), KCN3 (clone APC-101-F), SLAMF6 (clone 13G3), TIM3 (clone 5D12), TOX (clone TRX10), CD25 (clone PC61) and FoxP3 (clone MF23).

For the humanized PC-3 model, adherent cell line PC-3 (catalog no. CRL-1435, ATCC) was used. Human allogeneic PBMCs (AllCells) were used to purify negatively selected populations of T cells (catalog no. 19051, Stemcell) and CD11b monocytes (catalog no. 19058, Stemcell). Purified CD11b monocytes were cultured in ultra-low-attachment polystyrene plates (catalog no. D2650, Sigma-Aldrich) for 7 days. Next, 10 ng ml^–1^ GM-CSF (catalog no. 706-GR-050, R&D) and 20 ng ml^–1^ IL-4 (catalog no. 230-4R-025, R&D) were added. T cells were thawed and rested for 24 h with 1 ng ml^–1^ IL-2 (catalog no. 402-ML-20, R&D) 1 day before inoculation in mice. T cells, moDC and PC-3 cells were combined to deliver a SC injection of 1 × 10^7^ PC-3, 1 × 10^6^ T and 5 × 10^5^ moDC cells per NSG mouse (NOD.Cg-Prkdcscid Il2rgtm1Wjl/SzJ, 5–6-week-old female mice). For the HCT-116 model (catalog no. CCL-247, ATCC), NSG mice were inoculated with 1.0 × 10^6^ cells in the right hind flank (SC injection) and, at day 12 following inoculation, PBMCs (2.0 × 10^7^) were engrafted by IP inoculation. Treatment groups (*n* = 10) of 10 mg kg^–1^ isotype control and 16.1 mg kg^–1^ human anti-PD-1–GITR-L were prepared for IP injection. Measurement of tumor growth and data calculations were determined as previously described.

### PBMC bioactivity assays

PBMCs (2 × 10^5^ per well were plated in 96-well U-bottom plates with 0.5 µg ml^–1^ anti-human CD3. PBMCs from eight (proliferation) and five donors (cytokines) were tested using a serial dilution of treatment antibodies. After 48 and 96 h, supernatant was collected and analyzed for IFN-γ and IL-2 using an AlphaLISA kit. TNF-β was analyzed using a Milliplex kit. IFN-γ and IL-2 were measured using an EnSpire Alpha multimode plate reader. After 96 h, T cell proliferation was measured by ^3^H-thymidine incorporation as previously described. Stimulation index was calculated by dividing post-treatment proliferation and cytokine release by no treatment.

For the autologous MLR, dendritic cells (DCs) were derived by culture of 1 × 10^8^ plastic-adherent PBMCs with 4.8 µg of GM-CSF, 3 µg of IL-4 (7 days) and 12 ng of IL-1α and TNF-α (5 days). On day 7, DCs were harvested and irradiated for 7.3 min at 414 R min^–1^. Irradiated DCs (10^4^ per well) and purified CD4^+^ T cells (10^5^ per well) were added to 96-well U-bottom plates. Autologous MLR from seven PBMC donors was tested with a titration of treatment antibodies. After 5 days, supernatants were collected and analyzed for IFN-γ using AlphaLISA kits.

For antigen recall assay, 2 × 10^5^ PBMCs (*n* = 3) were used per well with 0.01 µg ml^–1^ CMVpp65. Cells were harvested on day 3 for CMVpp65 and assessed for T cell proliferation as previously indicated.

For T_reg_ suppression assay, T_regs_ were isolated using an EasySep CD4^+^CD25^+^CD127^low^ enrichment kit (catalog no. 18063, Stemcell). CD4^+^CD25^–^ T_eff_ cells were labeled with 0.25 µM CellTrace. The T_reg_ suppression assay was set up using a ratio of T_eff_:T_reg_ at 1:1 using 5 × 10^3^ cells of each type in a 96-well round-bottom plate. Anti-CD3 mAb OKT3 (catalog no. 16-0037-81, ThermoFisher) was also added to the wells for stimulation. After 5 days of incubation, cell proliferation was determined by flow cytometry as previously described. To determine the absolute number of carboxyfluorescein succinimidyl ester (CFSE)-labeled divided T_eff_ cells in each well, 20 µl of CountBright Absolute Counting Beads (ThermoFisher, catalog no. C36950) was added to each well. The absolute cell number of divided T_eff_ cells was determined using the following equation: ((no. of events in region containing CFSE-T_eff_ cells)/(no. of events in absolute count bead region)) × ((no. beads per test)/(test volume)).

### Rodent and cynomolgus monkey crossreactivity

For rodent crossreactivity, female Lewis strain rats and C57Bl/6 mice, all 8 weeks old, were purchased from Charles River Laboratories. Rat and mouse spleens were harvested and processed to create a single-cell suspension. Isolated splenocytes were added to plates coated with either 10 μg ml^–1^ anti-mouse CD3 (clone 145-2C11) or 10 μg ml^–1^ anti-rat CD3 (clone G4.18). Splenocytes were harvested at 48 and 72 h and cells processed for flow cytometry analysis. Cells were incubated with either Alexa Fluor 647-labeled anti-PD-1–GITR-L or isotype control and fluorescent-labeled antibodies: CD4 (clones RM4-5 and OX-35) and CD8a (clones 53-6.7 and OX-8). A total of 200,000 cynomolgus PD-1 and GITR (HEK293) cells were plated and incubated with a serial dilution of the treatment antibodies for binding affinity assessment. Cells were resuspended in 50 µl of a 1:100 diluted secondary R-Phycoerythrin AffiniPure F(ab’)_2_ Fragment Goat Anti-Human IgG.

The binding kinetics of anti-PD-1–GITR-L for cynomolgus PD-1, GITR and FcRn/β2m were determined by SPR, similarly to the method previously described. Recombinant extracellular domains of cynomolgus PD-1 and GITR were purchased from Creative Biomart (catalog nos. PDCD1-5223C and TNFRSF18-01C). The cyno FcRn/β2m heterodimer was internally generated.

Cynomolgus PBMCs were purchased from HumanCells Biosciences and treated with the NHP T cell activation/expansion kit (catalog no. 130-092-919, Miltenyi) in a 2:1 cell/bead ratio at 37 °C. Cells were stained with the following fluorescent-labeled antibiotics: CD3 (clone SP34-2), CD4 (clone L200), CD28 (clone CD28.2), CD95 (clone DX2) and CD8 (clone SK1). Cells were then pelleted and stained in 100 µl of concentration gradients of AF647-conjugated bispecific or isotype control. The frequency of anti-PD-1–GITR-L^+^ cells was analyzed on subsets of CD4^+^ T cell populations identified as central memory and effector memory T cells; central memory T cells were defined as CD28^+^/CD95^+^ while effector memory T cells were defined as CD28^–^/CD95^+^.

### Receptor copy number on activated PBMCs

Twenty million PBMCs were activated with 5 µg ml^–1^ PHA (catalog no. L8902, Sigma) and incubated with fluorescent-labeled antibody CCR7 (clone G043H7) before the addition of CD45RO (clone UCHL1), CD3 (clone SK7), CD4 (clone RPA-T4) and Alexa Fluor 647-labeled anti-GITR or PD-1. Cyno PBMCS were incubated with fluorescent-labeled antibodies CD3 (clone SP34-1), CD4 (clone L200), CD95 (clone DX2), CD28 (clone CD28.2) and Alexa Fluor 647-labeled anti-GITR or anti-PD-1. Quantum Simply Cellular anti-human IgG beads (catalog no. 816, Bangs Laboratories) were also stained with 25 µg ml^–1^ Alexa Fluor 647-labeled anti-GITR or anti-PD-1.

### Cynomolgus monkey PBMC bioactivity assays

Cynomolgus PBMCs (1 x 10^5^) were treated with 0.032 µg ml^–1^ anti-human CD3 (catalog no. 557052, BD) and incubated with either anti-PD-1–GITR-L or isotype mAb in a concentration gradient. After 2 days, cell proliferation was determined as previously described. For the NFκB reporter assay, 100,000 HEK 293-NFkB-cynoGITR^+^ cells were seeded in a 96-well flat-bottom plate with a serial dilution of treatment antibodies. After 24 h, luciferase activity was quantified with BriteLite Plus (catalog no. 6066761 PerkinElmer) and measured using an EnSpire Alpha multimode plate reader.

### PK/PD analysis in cynomolgus monkeys

PK and PD biomarker studies were conducted in cynomolgus monkeys (Charles River Laboratories). The procedure complied with all applicable sections of the Final Rules of the Animal Welfare Act regulations (Code of Federal Regulations, Title 9), the Public Health Service Policy on Humane Care and Use of Laboratory Animals from the Office of Laboratory Animal Welfare and the Guide for the Care and Use of Laboratory Animals from the National Research Council. Samples sizes were chosen empirically to ensure adequate statistical power, and were in line with field standards for techniques used in the study. Female cynomolgus monkeys (*Macaca fascicularis*), 2–4 years old (*n* = 3) were given a single IV bolus injection (2–5-min infusion) of anti-huPD-1-huGITR-L at the indicated doses. A placebo group (*n* = 2) was also included. Microbleed samples were taken at the indicated times. Plasma drug levels were determined by ELISA. Plates were coated with an anti-Id-PD-1 antibody and a biotinylated anti-huGITR-L antibody with SA-sulfo-TAG. The assay measured intact levels of anti-PD-1–GITR-L in plasma (3–5,000 ng m^–1^). Concentration–time data were analyzed using noncompartmental methods. PK parameters were calculated using WinNonlin Model 201 (WinNonlin5.2.1, Pharsight). Values of *R*^2^ or *R*^2^-adjusted ≥ 0.80 were required for acceptance of Lambda *z* (*λz*) estimates in noncompartmental analyses. Plasma samples for cytokine and chemokine analysis were collected at the indicated times and analyzed by MSD V-plex NHP 24-plex assay.

### Statistics and reproducibility

All experiments were repeated independently or performed with technical biological replicates as indicated in figure legends. In the case of human and cynomolgus monkey PBMCs, at least five donors were tested if not indicated differently. In animal studies, all treatment and control groups included about ten mice per group (specified in figure legends) and were randomized according to tumor volume at the start of treatment. Mice were randomly assigned without statistical predetermination of sample size. Blinding was not used in this study. Statistical analysis between groups was performed using Prism (v.8, GraphPad). Data are presented as means or median s.e.m., as stated in the figure legends. Statistical significance was determined as indicated in the figure legends, with *P* < 0.05 considered statistically significant. On principle, data were excluded for failed experiments only, the reasons for which included poor starting material and technical issues that could not be analyzed.

### Reporting Summary

Further information on research design is available in the Nature Research Reporting Summary linked to this article.

## Supplementary information

Supplementary Information Supplementary Tables 1–13.

Reporting Summary.

Supplementary Data 1 PDB structural validation report.

## Data Availability

Atomic coordinates and structure factors of the human GITR-L–GITR complex are deposited in PDB under accession code 7LAW. The NanoString gene expression and single-cell RNA-seq data that support the findings of this study have been deposited in the Gene Expression Omnibus under accession codes GSE189359 and GSE190105. Source data are provided with this paper. All other data supporting the findings of this study are available from the corresponding author on reasonable request.﻿

## Source data

Source Data Fig. 1 Numerical data for Fig. 1.
Source Data Fig. 2 Numerical data for Fig. 2.
Source Data Fig. 3 Numerical data for Fig. 3.
Source Data Fig. 4 Numerical data for Fig. 4.
Source Data Fig. 5 Numerical data for Fig. 5.
Source Data Fig. 6 Numerical data for Fig. 6.
Source Data Fig. 7 Numerical data for Fig. 7.
Source Data Fig. 8 Numerical data for Fig. 8.
Source Data Extended Data Fig. 3 Numerical data for ED Fig. 3.
Source Data Extended Data Fig. 4 Numerical data for ED Fig. 4.
Source Data Extended Data Fig. 5 Numerical data for ED Fig. 5.
Source Data Extended Data Fig. 6 Numerical data for ED Fig. 6.
Source Data Extended Data Fig. 7 Numerical data for ED Fig. 7.
Source Data Extended Data Fig. 8 Numerical data for ED Fig. 8.
Source Data Extended Data Fig. 9 Numerical data for ED Fig. 9.
