# Supplementary material for: An anti-PD-1–GITR-L bispecific agonist induces GITR clustering-mediated T cell activation for cancer immunotherapy
Source: Nat Cancer. 2022 Mar 7;3(3):337–54. doi: 10.1038/s43018-022-00334-9 (PMC8960412; doi:10.1038/s43018-022-00334-9)
Supplement: Supplementary file 1 — Supplementary Tables 1–13. [file 43018_2022_334_MOESM1_ESM.pdf]

nature portfolio

https://doi.org/10.1038/s43018-022-00334-9

Supplementary information

An anti-PD-1–GITR-L bispecific agonist induces GITR clustering-mediated T cell activation for cancer immunotherapy

In the format provided by the authors and unedited

Supplementary Tables

Supplementary Table 1. PD-1 and GITR expression on tumor infiltrated lymphocytes among different indications

Tumor Type	PD-1 (IHC ≥ 1)	GITR (IHC > 10%)
Colon	12/20	16/20
Pancreatic	7/19	8/19
Breast	6/10	5/19
Ovarian	13/20	4/20
Prostate	5/17	1/17
Lung	4/30	15/29
Renal	5/17	0/16
Melanoma	1/17	---

PD-1 IHC score criteria: IHC 1, 2 - 5 cells/20x at least 1/5 field, and GITR IHC score criteria: select 5 "hot spot" fields at 20X and use 5 discontinuous HPF (200X) per sample for scoring. Scores that ≥10% are considered positive in stroma area. Anti-PD-1 and anti-GITR staining on frozen tissues microarray samples.

Supplementary Table 2. Binding (EC_{50}) of anti-huPD-1-huGITR-L to human PD-1 and GITR

PD-1 and GITR transfected and endogenous expressing cell lines	Anti-PD-1-GITR-L EC_{50} (nM)	PD-1 EC_{50} (nM)	GITR-L EC_{50} (nM)
Human PD-1 on transfected HEK293 cells by Flow Cytometry[a]	0.7	0.6	---
Human GITR on transfected HEK293 cells by Flow Cytometry[a]	0.7	---	0.9
Human PD-1 and GITR on activated $CD4^+$ CM T cell by Flow Cytometry[b]	0.3	0.3	3.7
Human PD-1 and GITR on activated $CD4^+$ EM T cell by Flow Cytometry[b]	0.1	0.1	1.7

EC_{50} = half maximal effective concentration

a. Cell surface PD-1 and GITR expression detected using anti-PD-1-GITR-L, PD-1 and isotype-GITR-L on PD-1 and GITR HEK293 cell lines

b. Cell surface PD-1 and GITR expression detected using anti-PD-1-GITR-L, PD-1 and isotype-GITR-L on CD3/CD28 activated human PBMCs.

Supplementary Table 3. Binding kinetics of anti-huPD-1-huGITR-L to human recombinant PD-1 and GITR proteins

Molecule	Hu PD-1-His			Hu GITR-His		
	k_a (1/Ms)	k_d (1/s)	K_D (M)	k_a (1/Ms)	k_d (1/s)	K_D (M)
anti-PD-1	2.0E+05	5.3E-04	2.7E-09	---	---	---
GITR-L	---	---	---	1.1E+05	1.6E-03	1.5E-08
anti-PD-1-GITR-L	2.1E+05	5.0E-04	2.3E-09	1.1E+05	1.7E-03	1.6E-08

Supplementary Table 4. Anti-PD-1-GITR-L binding to human and cyno FcRn/β2m at pH 6.0

Molecules	huFcRn K_D (M)	cyFcRn K_D (M)
isotype-hIgG1-LALA	2.0E-06	1.7E-06
anti-PD-1-hIgG1-LALA	2.2E-06	1.9E-06
isotype/huGITR-L-hIgG1-LALA	2.4E-06	2.2E-06
Anti-PD-1-GITR-L-hIgG1-LALA	2.3E-06	2.0E-06

Supplementary Table 5. Total glycan occupancy of anti-huPD-1-huGITR-L following de-glycosylation with PNGase F and in vitro binding affinity and bioactivity screening of surrogate anti-muPD-1-muGITR-L bispecific

**Total glycan occupancy of anti-huPD-1-huGITR-L following de-glycosylation with PNGase F**	
Anti-PD-1-GITR-L (%)	Anti-PD-1-GITR-L + PNGase F (%)
91.9 %	0 %
23.2 %	6.1 %
65.3 %	5.1 %
6.5 %	6.5 %

**In vitro binding affinity and bioactivity screening of surrogate anti-muPD-1-muGITR-L bispecific**								
Molecules	Titer (mg/L)	% agg	PD-1 binding	GITR binding	T cell proliferation	IFNγ secretion	NFκB signaling	PD-L1 blocking
GITR-L	na	na	na	+++	+++	+++	+++	na

anti-PD-1	na	na	+++	na	na	na	na	+++
PD1-GITRL	16.7	2.1	+++	+++	+++	+++	+++	+++

Supplementary Table 6. Pharmacokinetic parameters of anti-muPD-1-muGITR-L in CT26 and EMT6 mouse syngeneic models

**CT26**			
Dose	$T_{1/2}$ (hour)	C_{max} (µg/mL)	AUC_{inf} (hr*µg/mL)
0.96 mg/kg	15	3	72
2.9 mg/kg	17	10	192
8.6 mg/kg	14	35	624
25.8 mg/kg	16	143	1632
**EMT6**			
Dose	$T_{1/2}$ (hour)	C_{max} (µg/mL)	AUC_{inf} (hr*µg/mL)
3.2 mg/kg	17	13	207
6..5 mg/kg	21	32	383
12.9 mg/kg	30	57	962
25.8 mg/kg	32	194	2038

Parameters were calculated using a non-compartmental model for IV administration

Supplementary Table 7. Mouse and human PD-1 and GITR expression by IHC of spleen and lymph nodes tissues from wild type (WT), heterozygous (HE) and homozygous (HO) GEMs

**Mouse and Human PD-1**					
**Gender**	**WT/Tg**	**Tissue**	**Anti HuPD-1**	**Anti MuPD-1**	**Rabbit IgG1**
Male	WT	Spleen	-	+	-
		LN	-	+	-
	WT	Spleen	-	+	-
		LN	-	+	-
Female	WT	Spleen	-	+	-
		LN	-	+	-

	WT	Spleen	-	+	-
		LN	-	+	-
Male	HO	Spleen	+	-	-
		LN	+	-	-
	HO	Spleen	+	-	-
		LN	+	-	-
Female	HO	Spleen	+	-	-
		LN	+	-	-
	HO	Spleen	+	-	-
		LN	+	-	-
Male	HE	Spleen	+	+	-
		LN	+	+	-
	HE	Spleen	+	+	-
		LN	+	+	-
Female	HE	Spleen	+	+	-
		LN	+	+	-

Mouse and Human GITR

Gender	WT/Tg	Tissue	Anti Hu GITR	Rabbit IgG1	Anti MuGITR	Rat IgG2b
Male	WT	Spleen	-	-	+	-
		LN	-	-	na	na
	WT	Spleen	-	-	+	-
		LN	-	-	na	na
Female	WT	Spleen	-	-	+	-
		LN	-	-	na	na
Male	HO	Spleen	±	-	-	-
		LN	+	-	na	na
	HO	Spleen	±	-	-	-
		LN	+	-	na	na
	HO	Spleen	-	-	-	-
		LN	+	-	na	na
Female	HO	Spleen	-	-	-	-
		LN	+	-	na	na
	HO	Spleen	-	-	-	-
		LN	+	-	na	na
Male	HE	Spleen	+	-	+	-
		LN	+	-	na	na
	HE	Spleen	+	-	+	-

		LN	+	-	na	na
Female	HE	Spleen	-	-	+	-
		LN	+	-	na	na

Supplementary Table 8. Percent sequence identity of human PD-1 and GITR in cynomolgus monkey

target	Human (%)	Cyno (%)
Human PDCD1	100	96
Human TNFRSF18	100	87

Supplementary Table 9. PD-1 and GITR expression and anti-PD-1-GITR-L cross-reactivity on normal human and cynomolgus monkey tissues by immuno-histochemistry

PD-1 and GITR expression on normal human and cynomolgus monkey tissues (IHC)						
Tissue type	Human PD-1 (% positive cells)	Cyno monkey PD-1 (% positive cells)	Human GITR (% positive cells)		Cyno monkey GITR (% positive cells)	
			ZPL2	ZPL3	I08762	I09447
Tonsil	3+	3+	5 – 20	21 – 50	21 – 50	> 50
Lymph Node	1+	2+	21 – 50	21 – 50	5 – 20	21 – 50
Spleen	+/-	2+	< 5	< 5	5 – 20	5 – 20
Stomach	2+ on lymphocytes	1+ on lymphatic nodule	< 5	< 5	5 – 20	21 – 50
Brain	0	0	0	0	0	0
Lung	0	0	0	0	0	0
Liver	0	0	0	0	0	0
Kidney	0	0	0	0	0	0
Colon	0	0	21 – 50	21 – 50	5 – 20	5 – 20
Pancreas	0	0	0	0	0	0
Muscle	0	0	0	0	0	0
Heart	0	0	0	0	0	0

Anti-PD-1-GITR-L cross-reactivity on normal human and cynomolgus monkey tissues (IHC)				
	Normal Human Tissue (% positive cells)		Normal Cynomolgus Primate (% positive cells)	
	ZPL2 (Female)	ZPL1 (Male)	I08493 (Male)	I08509 (Female)
Tonsil	21 – 50	21 – 50	> 50	> 50
Lymph Node	5 – 20	5 – 20	> 50	> 50
Spleen	5 – 20	Rare	21 – 50	> 50
Small intestine	< 5	5 – 20	Rare	< 5
Colon	5 – 20	< 5	0	< 5
Stomach	> 50	> 50	0	0
Pancreas	0	0	< 5 (lymphocytes staining)	< 5 (lymphocytes staining)
Lung	< 5 (lymphocytes staining)	0	0	0
Brain	0	0	0	0
Liver	0	0	0	0
Kidney	0	0	0	0
Muscle	0	0	0	0
Heart	0	0	0	0

Supplementary Table 10. Cross species reactivity binding of anti-huPD-1-huGITR-L

PD-1 and GITR transfected and endogenous expressing cell lines	Anti-PD-1-GITR-L EC_{50} (nM)
Cynomolgus PD-1 on transfected HEK293 cells[a]	3.40 nM
Cynomolgus GITR on transfected HEK293 cells[a]	0.49 nM
Cynomolgus PD-1 and GITR on activated $CD4^+$ CM T cells[b]	0.30 nM
Cynomolgus PD-1 and GITR on activated $CD4^+$ EM T cells[b]	1.0 nM
Rat PD-1 and GITR ($CD4^+$/$CD8^+$ T cells)	n/a
Mouse PD-1 and GITR ($CD4^+$/$CD8^+$ T cells)	n/a

EC_{50} = half maximal effective concentration maximal effective concentration

a. Cell surface PD-1 and GITR expression detected using anti-PD-1-GITR-L on PD-1 and GITR HEK293 cell lines

b. Cell surface PD-1 and GITR expression detected using anti-PD-1-GITR-L on CD3/CD28 activated cynomolgus PBMCs.

Supplementary Table 11. Binding kinetics of anti-huPD-1-huGITR-L to human and cynomolgus recombinant PD-1 and GITR proteins

Molecule	Hu PD-1-His			Cyno PD-1-His		
	k_a (1/Ms)	k_d (1/s)	K_D (M)	k_a (1/Ms)	k_d (1/s)	K_D (M)
Anti-PD-1-GITR-L	3.0E+05	5.9E-04	2.0E-09	3.2E+05	1.2E-03	3.7E-09
	Hu GITR-His			Cyno GITR-His		
	k_a (1/Ms)	k_d (1/s)	K_D (M)	k_a (1/Ms)	k_d (1/s)	K_D (M)
	5.6E+05	1.0E-03	1.8E-09	1.1E+06	8.7E-04	7.9E-10

Supplementary Table 12. Pharmacokinetic parameters of anti-huPD-1-huGITR-L in cynomolgus monkey

Dose (mg/kg)	Assay Type	$T_{1/2}$ (hr)	C_{max} (µg/mL)	AUC_{inf} (hour*µg/mL)	AUC_{inf}/D	CL (mL/hour/kg)	Vss (mL/kg)
0.1	Intact	5.84	0.77	2.4	24	41.6	174
1	Intact	9.28	13	41	41	25.5	205
10	Intact	13.5	286	813	81	12.3	93
30	Intact	18.4	890	3140	105	12.1	86

Parameters were calculated using a non-compartmental model for IV administration

Supplementary Table 13. X-ray data collection and refinement statistics for human GITR-GITR-L complex

Data in parameters correspond to highest-resolution shells. Following standard abbreviations were used: number (No), asymmetric unit (AU), root-mean-square (r.m.s).

**Data collection**	
Resolution (Å)	2.75
Wavelength (Å)	1.000
Space Group	I23
Cell dimensions<br>a,b,c (Å)<br>α,β,γ (°)	172.7, 172.7, 172.7<br>90, 90, 90
Molecules in AU	two GITR-GITRL
Total reflections	434586

Unique reflections	21565
Multiplicity	20.2
Completeness spherical (%)	97
Mean I/ δ (I)	19.1
Rpim (%)	3.6
CC1/2	0.99
**Refinement**	
Rcryst (%)	22.9
Rfree (%)	24.6
R.m.s. deviations<br>Bond lengths (Å)<br>Bond angles (°)	0.008<br>1.08
Ramachandran (%)<br>Favored, allowed, outliers	92.1, 6.0, 1.9
No. atoms<br>Protein<br>Water	3284<br>48
B-factors<br>Protein<br>Water	80.4<br>71.3
PDB ID	7LAW
