# Supplementary material for: An anti-PD-1–GITR-L bispecific agonist induces GITR clustering-mediated T cell activation for cancer immunotherapy
Source: Nat Cancer. 2022 Mar 7;3(3):337–54. doi: 10.1038/s43018-022-00334-9 (PMC8960412; doi:10.1038/s43018-022-00334-9)
Supplement: Supplementary file 2 — Reporting Summary. [file 43018_2022_334_MOESM2_ESM.pdf]

Corresponding author(s): Hamsell M. Alvarez

Last updated by author(s): November 11th, 2021

Reporting Summary

Nature Research wishes to improve the reproducibility of the work that we publish. This form provides structure for consistency and transparency in reporting. For further information on Nature Research policies, see our Editorial Policies and the Editorial Policy Checklist.

Statistics

For all statistical analyses, confirm that the following items are present in the figure legend, table legend, main text, or Methods section.

n/a	Confirmed	
☐	☒	The exact sample size (*n*) for each experimental group/condition, given as a discrete number and unit of measurement
☐	☒	A statement on whether measurements were taken from distinct samples or whether the same sample was measured repeatedly
☐	☒	The statistical test(s) used AND whether they are one- or two-sided<br>*Only common tests should be described solely by name; describe more complex techniques in the Methods section.*
☒	☐	A description of all covariates tested
☒	☐	A description of any assumptions or corrections, such as tests of normality and adjustment for multiple comparisons
☐	☒	A full description of the statistical parameters including central tendency (e.g. means) or other basic estimates (e.g. regression coefficient) AND variation (e.g. standard deviation) or associated estimates of uncertainty (e.g. confidence intervals)
☐	☒	For null hypothesis testing, the test statistic (e.g. *F*, *t*, *r*) with confidence intervals, effect sizes, degrees of freedom and *P* value noted<br>*Give P values as exact values whenever suitable.*
☒	☐	For Bayesian analysis, information on the choice of priors and Markov chain Monte Carlo settings
☒	☐	For hierarchical and complex designs, identification of the appropriate level for tests and full reporting of outcomes
☒	☐	Estimates of effect sizes (e.g. Cohen's *d*, Pearson's *r*), indicating how they were calculated

Our web collection on statistics for biologists contains articles on many of the points above.

Software and code

Policy information about availability of computer code

Data collection	LSR Fortessa, BD FACSymphony A3 flow cytometer. Nanostring data analysis was done using Nanostring propietary software (Nsolver 4.0). Single cell RNA sequencing using chromium 3'V3 chemistry (10X Genomics).
Data analysis	BD FACSDiva 9.0, Flowjo 10.4, Prism 8, Phaser 2.8, REFMAC5.5, AutoBUSTER 2.10, Pymol 2.5, Ximpp 2.0, WinNonlin 5.2.1, CellRanger 3.0.2, Loupe cell browser 3.1.1, Seurat 4.0, HALO 2.0, StudyLog 2.1.1, Immunospot 5.1, CCP4 7.1, COOT 0.5, Scipion 3.0.0

For manuscripts utilizing custom algorithms or software that are central to the research but not yet described in published literature, software must be made available to editors and reviewers. We strongly encourage code deposition in a community repository (e.g. GitHub). See the Nature Research guidelines for submitting code & software for further information.

Data

Policy information about availability of data

All manuscripts must include a data availability statement. This statement should provide the following information, where applicable:

- Accession codes, unique identifiers, or web links for publicly available datasets
- A list of figures that have associated raw data
- A description of any restrictions on data availability

Atomic coordinates and structure factors of the human GITR-L-GITR complex are deposited in the Protein Data Bank (PDB) under accession code 7LAW. The Nanostring gene expression and single-cell RNA-seq data that support the findings of this study have been deposited in the Gene Expression Omnibus (GEO) under accession codes GSE189359 and GSE190105. Source data have been provided as Source Data files. All other data supporting the findings of this study are available from the corresponding author on reasonable request.

Field-specific reporting

Please select the one below that is the best fit for your research. If you are not sure, read the appropriate sections before making your selection.

☒ Life sciences ☐ Behavioural & social sciences ☐ Ecological, evolutionary & environmental sciences

For a reference copy of the document with all sections, see nature.com/documents/nr-reporting-summary-flat.pdf

Life sciences study design

All studies must disclose on these points even when the disclosure is negative.

Sample size	In case of human or cynomolgus monkey PBMCs, at least five donors were tested if not indicated differently. In animal studies, all treatment and control groups had about 10 mice per group (specified in figure legends). Samples sizes were chosen empirically to ensure adequate statistical power and were in the line with field standards for techniques used in the study (PMID: 31974274, 31036879, 28446565, 28069723, 30389797, 28405505)
Data exclusions	No data were excluded from the analysis.
Replication	Experiments were repeated independently or performed with technical biological replicates. The precise number of repeats is provided in the figure legend.
Randomization	In animal studies, all treatment and control groups were randomized according to tumor volume at the start of treatment. Samples for in vitro immunological assays were randomly assigned.
Blinding	Blinding was not used in this study (not possible for studies based on treatment and general conditions of the samples used).

Reporting for specific materials, systems and methods

We require information from authors about some types of materials, experimental systems and methods used in many studies. Here, indicate whether each material, system or method listed is relevant to your study. If you are not sure if a list item applies to your research, read the appropriate section before selecting a response.

Materials & experimental systems

n/a	Involved in the study	
☐	☒	Antibodies
☐	☒	Eukaryotic cell lines
☒	☐	Palaeontology and archaeology
☐	☒	Animals and other organisms
☒	☐	Human research participants
☒	☐	Clinical data
☒	☐	Dual use research of concern

Methods

n/a	Involved in the study	
☒	☐	ChIP-seq
☐	☒	Flow cytometry
☒	☐	MRI-based neuroimaging

Antibodies

Antibodies used	For human binding studies: HRP-sheep anti human Clq 1/100 (Cat.# ab46191, Abcam), R-Phycoerythrin AffiniPure F(ab')2 Fragment Goat Anti-Human and mouse lgG, Fcg and Fab fragment specific 1/100 (Cat. # 109-116-1 & Cat. # 109-116-097, Jackson lmmunoResearch). For mouse studies : CD45 APC-Cy7 1/50 (clone30-Fll, Cat.# 559864, BD Bioscience), CD4 PerCp Cy5 1/100 (clone RM4.5, Cat.# 561090, BD Bioscience), CD8 PE-Cy7 1/30 (clone 53-6.7, Cat.# 561097, BD Bioscience), CD62L FITC 1/100 (clone MEL-14, Cat.# 553150, BD Bioscience), ICOS BV421 1/50 (clone 7E.17G9, Cat# 564070, BD Bioscience), CD44 BV786 1/100 (clone IM7, Cat.# 563736, BD Bioscience), TIGIT BV510 1/100 (clone 1G9, Cat.# 142103, Biolegend), Ki67 PE 1/50 (clone 16A8, Cat.#. 652403, Biolegend), CD25 PE 1/50 (clone PC61, Cat.# 553866, BD Bioscience), FoxP3 AF-647 1/100 (clone MF23, Cat.# 560401, BD Bioscience), CD4 APC-Cy7 1/50 (clone RM4-5, Cat.# 565650, BD Bioscience), TIGIT BV421 1/50 (clone 1G9, Cat.# 565270, BD Bioscience), CD62L BV510 1/100 (clone MEL-14, Cat.# 563117, BD Bioscience), Ki67 AF488 1/100 (clone 11F6, Cat.#151204, Biolegend), ICOS PE 1/100 (clone 7E.17G9, Cat.# 552146, BD Bioscience), CD226 PE 1/100 (clone 10E5, Cat.# 128809, Biolegend), KCN3 FITC 1/100 (clone APC-101-F, Cat.# PAl-12654, Alomone Labs), SLAMF6 BV421 1/100 (clone 13G3, Cat# 740090, BD Bioscience), TIM3 BV650 1/50 (clone 5D12, Cat.# 747623, BD Bioscience), CD25 BV605 1/50 (clone PC61, Cat.# 563061, BD Bioscience), TOX EF660 1/200 (clone TRX510, cat. # 50-6502-82, EBioscience), GZMB PE 1/100 (clone GB11, Cat. # MA1-80734, ThermoFisher) and CD3 10 ugs/ml (Clone 145-2C11, cat. # 16-0031-86, e-Bioscience). For cyno studies: CD3 V500 1/25 (clone SP34-2, cat.# 560770, BD Bioscience), CD4 APC 1/100 (clone L200, cat.# 564107, BD Bioscience), CD28 PerCp 1/50 (clone CD28.2 cat.# 562613, BD Bioscience), CD95 FITC 1/20 (clone DX2, cat.# 561636, BD Bioscience), and CD3 BV421 1/100 (clone SKl, cat.# 344716, Biolegend). For rat studies: CD3 10 ugs/ml (clone G4.18, cat.# 554829, BD Bioscience), CD4 PE 1/50 (clone OX-35, cat. # 12-0040-82, EBioscience), Cd8a AF488 1/100 (clone OX-8, cat. # ab256296, Abcam). For human studies: CCR7 PE/Cyanine7 1/50 (clone G043H7, cat. # 353255, Biolegend), CD45RO APC 1/100 (clone UCHL1, cat. # 33304210, Biolegend), CD3 FITC 1/100 (clone Sk7, cat. # 344803, Biolegend), CD4 PE 1/50 (clone RPA-T4, cat. # 300507, Biolegend), CD3 1/100 (clone OKT3, cat. # 16-003781, EBioscience). For IHCstudies: Mouse or rabbit anti-huGITR lgG2b (0.38 ug/ml, clone AGGIE.11, AbbVie or 2.5 ug/ml, clone D919D, Cat.,# 680145, Cell Signaling), anti-huPD-1 lgG2b antibodies (0.63 ug/ml clone 12All, AbbVie), Rabbit anti-human GITR lgGl (5 ug/ml, clone EPR20566, Cat.# ab223841, Abcam), rat anti-mouse GITR lgGl (5 ug/ml, clone DTA-1, Cat.# ab210258, Abcam), Rabbit anti-human PD-1 lgGl (1 ug/ml, clone EPR48772, Cat.# ab137132), rat anti-mouse PD-1 lgGl (5ug/ml, clone EPR 20665, Cat.# ab214421, Abcam), and FoxP3 (2 ug/ml, clone D2W8E, Cat. # 987377, Cell Signaling).
Validation	All antibodies are from commercial sources and have been validated by the vendors and validation materials are available on the appropriate websites from vendors (Abcam, Jackson Immunoresearch, BD Bioscience, Biolegend, AlomeLabs, and CellSignaling). For flowcyometry, antibodies were used as suggested by the commercial vendors and finally determined by experimental optimization.

Eukaryotic cell lines

Policy information about cell lines

Cell line source(s): Human, mouse and cyno PD-1 and GITR HEK293 cells (AbbVie), human PD-1 expressing NFAT reporter Jurkat cell line (cat.# CS187102, Promega), Human PD-Ll expressing CHOKl activator cells (cat.# CS187108, Propagation model, Promega), Human, mouse and cyno PD-1 and GITR NFkBHEK293 cells (AbbVie), CT26 (cat.# CRL-2638, ATCC), JC (cat.# CRL-2116, ATCC), EMT6 (cat.# CRL-2755, ATCC), MC-38 (University of Chicago, license agreement#L-085-2016/0 with the NIH), PC-3 (cat.# CRL-1435, ATCC), HCT-116 (cat.# CCL-247, ATCC), B16F10 (cat. # CRL-6475, ATCC).

Authentication: Cells did no undergo complete authentication following receipt from the indicated sources. However, cell lines purchased from vendors including ATCC were authenticated prior to purchase, and once in house, were rapidly expanded, cryopreserved at early passages, and handled by a limited number of trained and skilled individuals.

Mycoplasma contamination: Stocks for cell lines in the described studies were deemed free of mycoplamsa contamination following testing using standard commercially kits (ie, Lonza MycoAlert Plus).

Commonly misidentified lines (See ICLAC register): No commonloy misidentfied lines were used.

Animals and other organisms

Policy information about studies involving animals; ARRIVE guidelines recommended for reporting animal research

Laboratory animals: Mouse studies: Five- to six-week-old wild type Balb/c and eigth-week C57BL/6 female mice were obtained from Taconic (Oxnard, CA) or Charles River Laboratory. NSG (NOD.Cg-Prkdcscid Il2rgtm1Wjl/SzJ) five-to six-week-old mix gender mice were obtained from Charles River Laboratory. Eight-week-old wild type female Lewis rats were obtained from Charles River Laboratory. For human GITR and PD-1 GEM: Generation, and injection of ES cell clones into blastocysts, chimera generation and breeding, and germline transmission screening were performed by GenOway. Breeding of C57BL/6 homozygous PD-1 and GITR mice was performed by Charles River Laboratories. PK and PD biomarker studies were conducted in 2-4-years-old female cynomolgus monkey (Macaca fascicularis) with assistance of Charles River Laboratories (Mattawan, MI). Rodents were housed in an environment with temperature ranging from 68-76 degrees Fahrenheit, humidity of 30-45%, and a light cycle with 14 hours of light and 10 hours of dark.

Wild animals: No wild animals were used in the study.

Field-collected samples: This study did not include samples collected from the field.

Ethics oversight: Mice and rats were housed under specific pathogen-free conditions in an AAALAC, International (Frederick, MD) accredited facility. All mouse and rats procedures were performed in accordance with protocols approved by the Global Animal Welfare internal Institutional Animal Care and Use Committee (IACUC) and were performed in accordance with the guidelines in the Guide for the Care and Use of Laboratory Animals (National Resource Council, 2018). For cynos: The procedure complies with all applicable sections of the Final Rules of the Animal Welfare Act regulations (Code of Federal Regulations, Title 9), the Public Health Service Policy on Humane Care and Use of Laboratory Animals from the Office of Laboratory Animal Welfare, and the Guide for the Care and Use of Laboratory Animals from the National Research Council.

Note that full information on the approval of the study protocol must also be provided in the manuscript.

Flow Cytometry

Plots

Confirm that:

☒ The axis labels state the marker and fluorochrome used (e.g. CD4-FITC).

☒ The axis scales are clearly visible. Include numbers along axes only for bottom left plot of group (a 'group' is an analysis of identical markers).

☒ All plots are contour plots with outliers or pseudocolor plots.

☒ A numerical value for number of cells or percentage (with statistics) is provided.

Methodology

Sample preparation: Human peripheral blood mononuclear cells were isolated from human donors by Ficoll separation, and Cynomolgus PBMCs were purchased from Human Cells Biosciences and treated with non-human primate T cell activation/expansion kit (cat. # 130-092-919, Miltenyi Biotec).
Mouse TDLN were processed individually by gently macerating between two frosted micro slides and pipetting up and down to release cells into the media thoroughly. To obtain single-cell suspensions, tumors were mechanically minced into ~ 2 mm fragments followed by dissociation using a gentleMACS dissociator system in RPMI plus 10 % FBS with of 2.0 µg/ml collagenase A, and 0.1 µg/ml DNAse I at 37oC for 40 minutes. The digest was filtered through a 40-µm cell strainer to remove macroscopic debris, and the final cell preparation was washed with phosphate-buffered saline plus 1% fetal bovine serum.

Instrument: BD FACSymphony A3 flow cytometer, and LSR Fortessa (BD Biosciences, San Jose, CA).

Software: BD FACSDiva, Flowjo10.4

Cell population abundance: After sorting, sorted cells were re-run with the exact setting on the same instrument. Sorted cells with a purity of > 90% were subject to the following experiments.

Gating strategy

The gating strategy for analysis of mouse PD markers is as follows: First, cells are gated as singlets by FSC or SSC to remove doublets. Next, a celullar size gate using SSC and FSC removes debris. Viable cells are then gated based on a Live/Dead stain. Finally, lymphocytes are gated as CD45+, then CD3+ for T cells (including CD4 and CD8+ T cells), CD4+CD25+FoxP3+ for Tregs, CD62L and CD44 for memory T cells, ki67 for cell proliferation, ICOS, TIGIT, CD226 and KCNA3 for T cell activation , SLAMF6 and TIM3 for exhausted and progenitor T cells. Similar gating strategy was used for human and cyno memory T cells: CCR7 and CD45RA for human and CD95 and CD28 for cynos.

☒ Tick this box to confirm that a figure exemplifying the gating strategy is provided in the Supplementary Information.
