# Supplementary material for: An anti-PD-1–GITR-L bispecific agonist induces GITR clustering-mediated T cell activation for cancer immunotherapy
Source: Nat Cancer. 2022 Mar 7;3(3):337–54. doi: 10.1038/s43018-022-00334-9 (PMC8960412; doi:10.1038/s43018-022-00334-9)
Supplement: Supplementary file 3 — PDB structural validation report. [file 43018_2022_334_MOESM3_ESM.pdf]

Preliminary Full wwPDB X-ray Structure Validation Report (i)

Jan 6, 2021 – 01:48 PM EST

Deposition ID : D_1000253991

This is a Preliminary Full wwPDB X-ray Structure Validation Report.

This report is produced by the wwPDB Deposition System during initial deposition but before annotation of the structure.

We welcome your comments at *validation@mail.wwpdb.org*
A user guide is available at
https://www.wwpdb.org/validation/2017/XrayValidationReportHelp
with specific help available everywhere you see the (i) symbol.

PRELIMINARY VALIDATION REPORT

The following versions of software and data (see references (i)) were used in the production of this report:

MolProbity	:	4.02b-467
Mogul	:	1.8.5 (274361), CSD as541be (2020)
Xtriage (Phenix)	:	1.13
EDS	:	2.16
Percentile statistics	:	20191225.v01 (using entries in the PDB archive December 25th 2019)
Refmac	:	5.8.0158
CCP4	:	7.0.044 (Gargrove)
Ideal geometry (proteins)	:	Engh & Huber (2001)
Ideal geometry (DNA, RNA)	:	Parkinson et al. (1996)
Validation Pipeline (wwPDB-VP)	:	2.16

1 Overall quality at a glance (i)

The following experimental techniques were used to determine the structure:
X-RAY DIFFRACTION

The reported resolution of this entry is 2.75 Å.

Percentile scores (ranging between 0-100) for global validation metrics of the entry are shown in the following graphic. The table shows the number of entries on which the scores are based.

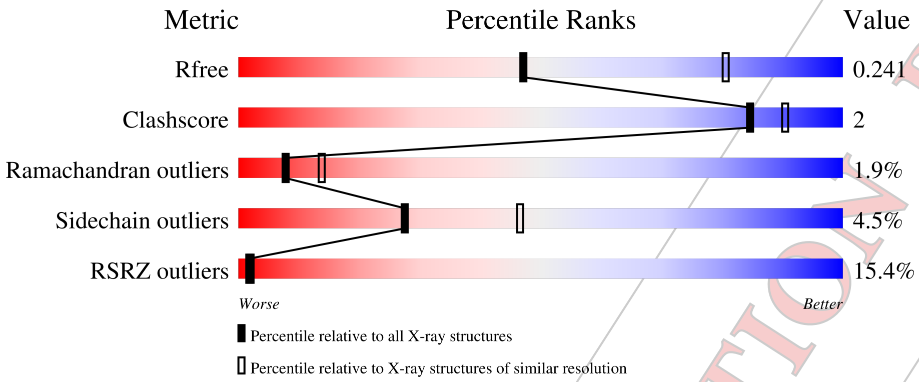

Metric	Whole archive (#Entries)	Similar resolution (#Entries, resolution range(Å))
R_{free}	130704	1235 (2.78-2.74)
Clashscore	141614	1277 (2.78-2.74)
Ramachandran outliers	138981	1257 (2.78-2.74)
Sidechain outliers	138945	1257 (2.78-2.74)
RSRZ outliers	127900	1207 (2.78-2.74)

The table below summarises the geometric issues observed across the polymeric chains and their fit to the electron density. The red, orange, yellow and green segments of the lower bar indicate the fraction of residues that contain outliers for >=3, 2, 1 and 0 types of geometric quality criteria respectively. A grey segment represents the fraction of residues that are not modelled. The numeric value for each fraction is indicated below the corresponding segment, with a dot representing fractions <=5% The upper red bar (where present) indicates the fraction of residues that have poor fit to the electron density. The numeric value is given above the bar.

Mol	Chain	Length	Quality of chain
1	A	128	16% / 90% 5% • 5%
1	B	128	9% / 88% 8% 5%
2	R	147	14% / 54% 11% 35%
2	S	147	10% / 56% 10% 35%

PRELIMINARY VALIDATION REPORT

2 Entry composition (i)

There are 4 unique types of molecules in this entry. The entry contains 3388 atoms, of which 0 are hydrogens and 0 are deuteriums.

In the tables below, the ZeroOcc column contains the number of atoms modelled with zero occupancy, the AltConf column contains the number of residues with at least one atom in alternate conformation and the Trace column contains the number of residues modelled with at most 2 atoms.

- Molecule 1 is a protein.

Mol	Chain	Residues	Atoms					ZeroOcc	AltConf	Trace
			Total	C	N	O	S			
1	A	122	974	628	160	181	5	0	0	0
1	B	122	974	628	160	181	5	0	0	0

- Molecule 2 is a protein.

Mol	Chain	Residues	Atoms					ZeroOcc	AltConf	Trace
			Total	C	N	O	S			
2	R	96	668	409	123	125	11	0	0	0
2	S	96	668	409	123	125	11	0	0	0

- Molecule 3 is 2-acetamido-2-deoxy-beta-D-glucopyranose (three-letter code: NAG) (formula: $C_8H_{15}NO_6$).

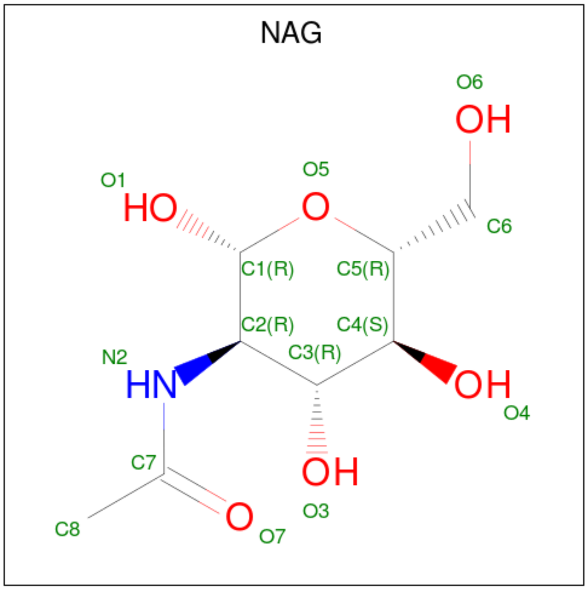

VALIDATION REPORT

Mol	Chain	Residues	Atoms				ZeroOcc	AltConf
			Total	C	N	O		
3	A	1	14	8	1	5	0	0
3	B	1	14	8	1	5	0	0
3	R	1	14	8	1	5	0	0
3	S	1	14	8	1	5	0	0

- Molecule 4 is water.

Mol	Chain	Residues	Atoms		ZeroOcc	AltConf
			Total	O		
4	W	48	48	48	0	0

PRELIMINARY VALIDATION REPORT

3 Residue-property plots (i)

These plots are drawn for all protein, RNA, DNA and oligosaccharide chains in the entry. The first graphic for a chain summarises the proportions of the various outlier classes displayed in the second graphic. The second graphic shows the sequence view annotated by issues in geometry and electron density. Residues are color-coded according to the number of geometric quality criteria for which they contain at least one outlier: green = 0, yellow = 1, orange = 2 and red = 3 or more. A red dot above a residue indicates a poor fit to the electron density (RSRZ > 2). Stretches of 2 or more consecutive residues without any outlier are shown as a green connector. Residues present in the sample, but not in the model, are shown in grey.

- Molecule 1:

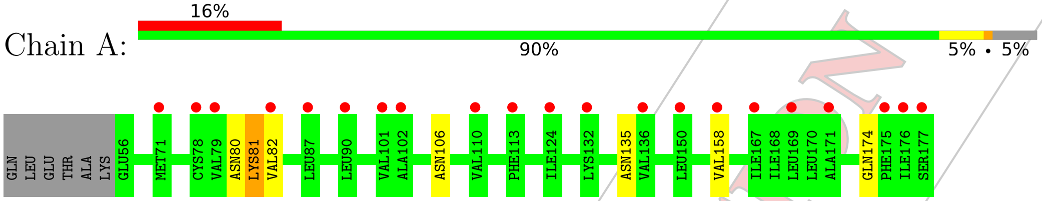

- Molecule 1:

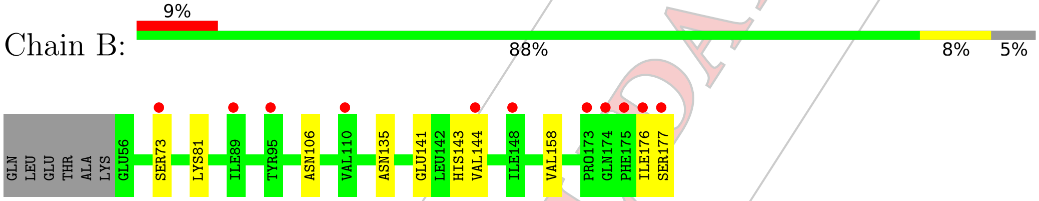

- Molecule 2:

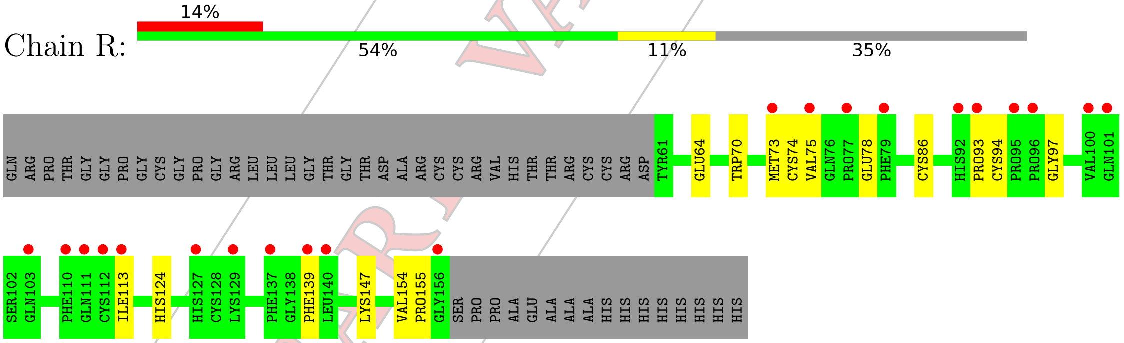

- Molecule 2:

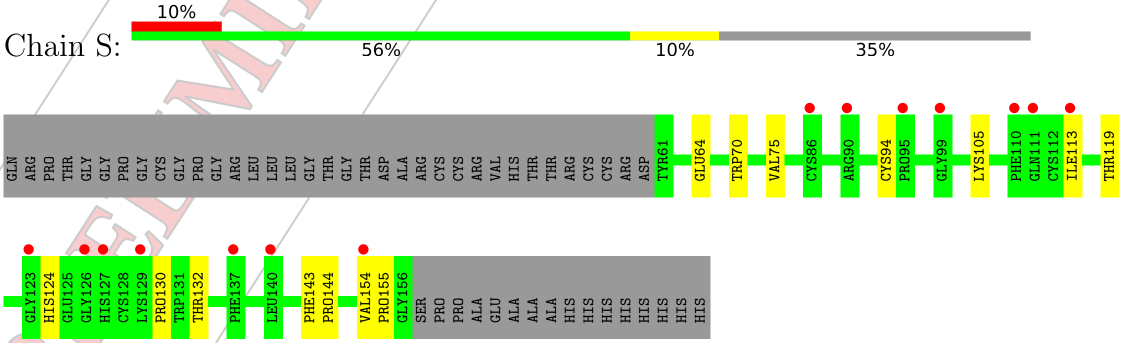

PRELIMINARY VALIDATION REPORT

4 Data and refinement statistics (i)

Property	Value	Source
Space group	I 2 3	Depositor
Cell constants<br>a, b, c, α, β, γ	172.66Å 172.66Å 172.66Å<br>90.00° 90.00° 90.00°	Depositor
Resolution (Å)	122.09 – 2.75<br>122.09 – 2.93	Depositor<br>EDS
% Data completeness<br>(in resolution range)	96.5 (122.09-2.75)<br>100.0 (122.09-2.93)	Depositor<br>EDS
R_{merge}	(Not available)	Depositor
R_{sym}	(Not available)	Depositor
$< I/\sigma(I) >$ [1]	1.86 (at 2.91Å)	Xtriage
Refinement program	BUSTER 2.11.7 (18-SEP-2020)	Depositor
R, R_{free}	0.229 , 0.236<br>0.232 , 0.241	Depositor<br>DCC
R_{free} test set	968 reflections (5.22%)	wwPDB-VP
Wilson B-factor (Å^2)	81.0	Xtriage
Anisotropy	0.000	Xtriage
Bulk solvent k_{sol}(e/Å^3), B_{sol}(Å^2)	0.34 , 61.4	EDS
L-test for twinning[2]	$< \|L\| > = 0.51$, $< L^2 > = 0.34$	Xtriage
Estimated twinning fraction	0.021 for -l,-k,-h	Xtriage
F_o,F_c correlation	0.91	EDS
Total number of atoms	3388	wwPDB-VP
Average B, all atoms (Å^2)	80.0	wwPDB-VP

Xtriage's analysis on translational NCS is as follows: *The largest off-origin peak in the Patterson function is 2.55% of the height of the origin peak. No significant pseudotranslation is detected.*

[1] Intensities estimated from amplitudes.

[2] Theoretical values of $< |L| >$, $< L^2 >$ for acentric reflections are 0.5, 0.333 respectively for untwinned datasets, and 0.375, 0.2 for perfectly twinned datasets.

PRELIMINARY VALIDATION REPORT

5 Model quality (i)

5.1 Standard geometry (i)

Bond lengths and bond angles in the following residue types are not validated in this section: NAG

The Z score for a bond length (or angle) is the number of standard deviations the observed value is removed from the expected value. A bond length (or angle) with $|Z| > 5$ is considered an outlier worth inspection. RMSZ is the root-mean-square of all Z scores of the bond lengths (or angles).

Mol	Chain	Bond lengths		Bond angles	
		RMSZ	#$\|Z\|$ >5	RMSZ	#$\|Z\|$ >5
1	A	0.41	0/999	0.64	0/1360
1	B	0.43	0/999	0.64	0/1360
2	R	0.40	0/691	0.63	0/933
2	S	0.40	0/691	0.63	0/933
All	All	0.41	0/3380	0.64	0/4586

There are no bond length outliers.

There are no bond angle outliers.

There are no chirality outliers.

There are no planarity outliers.

5.2 Too-close contacts (i)

In the following table, the Non-H and H(model) columns list the number of non-hydrogen atoms and hydrogen atoms in the chain respectively. The H(added) column lists the number of hydrogen atoms added and optimized by MolProbity. The Clashes column lists the number of clashes within the asymmetric unit, whereas Symm-Clashes lists symmetry-related clashes.

Mol	Chain	Non-H	H(model)	H(added)	Clashes	Symm-Clashes
1	A	974	0	961	3	0
1	B	974	0	961	6	0
2	R	668	0	555	5	0
2	S	668	0	555	5	0
3	A	14	0	13	0	0
3	B	14	0	13	0	0
3	R	14	0	13	0	0
3	S	14	0	13	0	0
4	W	48	0	0	1	0
All	All	3388	0	3084	15	0

PRELIMINARY VALIDATION REPORT

The all-atom clashscore is defined as the number of clashes found per 1000 atoms (including hydrogen atoms). The all-atom clashscore for this structure is 2.

All (15) close contacts within the same asymmetric unit are listed below, sorted by their clash magnitude.

Atom-1	Atom-2	Interatomic distance (Å)	Clash overlap (Å)
1:B:143:HIS:CE1	1:B:176:ILE:HG23	2.40	0.56
2:R:139:PHE:CZ	2:S:132:THR:HG21	2.42	0.55
1:B:143:HIS:HE1	1:B:176:ILE:HG23	1.76	0.51
1:A:81:LYS:HD2	1:B:177:SER:HA	1.93	0.50
1:A:82:VAL:HG22	1:B:144:VAL:HG11	1.92	0.50
2:R:97:GLY:HA2	2:R:147:LYS:O	2.13	0.49
2:R:94:CYS:O	2:R:124:HIS:HA	2.13	0.49
2:S:94:CYS:O	2:S:124:HIS:HA	2.14	0.48
1:B:143:HIS:CE1	1:B:176:ILE:CG2	2.99	0.45
2:S:119:THR:HA	2:S:130:PRO:HA	2.00	0.44
2:R:155:PRO:HD2	2:S:155:PRO:HG3	1.99	0.44
2:R:78:GLU:HG2	2:R:93:PRO:HB3	2.00	0.42
1:A:174:GLN:HG2	4:W:54:HOH:O	2.19	0.41
2:S:143:PHE:CD1	2:S:144:PRO:HD2	2.55	0.41
1:B:141:GLU:HG3	1:B:176:ILE:HD13	2.03	0.41

There are no symmetry-related clashes.

5.3 Torsion angles (i)

5.3.1 Protein backbone (i)

In the following table, the Percentiles column shows the percent Ramachandran outliers of the chain as a percentile score with respect to all X-ray entries followed by that with respect to entries of similar resolution.

The Analysed column shows the number of residues for which the backbone conformation was analysed, and the total number of residues.

Mol	Chain	Analysed	Favoured	Allowed	Outliers	Percentiles	
1	A	120/128 (94%)	115 (96%)	5 (4%)	0	100	100
1	B	120/128 (94%)	116 (97%)	3 (2%)	1 (1%)	19	34
2	R	94/147 (64%)	82 (87%)	7 (7%)	5 (5%)	2	2
2	S	94/147 (64%)	81 (86%)	11 (12%)	2 (2%)	7	12
All	All	428/550 (78%)	394 (92%)	26 (6%)	8 (2%)	8	14

PRELIMINARY VALIDATION REPORT

All (8) Ramachandran outliers are listed below:

Mol	Chain	Res	Type
2	R	64	GLU
2	R	74	CYS
2	S	64	GLU
1	B	73	SER
2	R	70	TRP
2	S	70	TRP
2	R	73	MET
2	R	86	CYS

5.3.2 Protein sidechains ⓘ

In the following table, the Percentiles column shows the percent sidechain outliers of the chain as a percentile score with respect to all X-ray entries followed by that with respect to entries of similar resolution.

The Analysed column shows the number of residues for which the sidechain conformation was analysed, and the total number of residues.

Mol	Chain	Analysed	Rotameric	Outliers	Percentiles	
1	A	109/114 (96%)	104 (95%)	5 (5%)	27	46
1	B	109/114 (96%)	105 (96%)	4 (4%)	34	54
2	R	69/120 (58%)	66 (96%)	3 (4%)	29	48
2	S	69/120 (58%)	65 (94%)	4 (6%)	20	35
All	All	356/468 (76%)	340 (96%)	16 (4%)	27	46

All (16) residues with a non-rotameric sidechain are listed below:

Mol	Chain	Res	Type
1	A	80	ASN
1	A	81	LYS
1	A	106	ASN
1	A	135	ASN
1	A	158	VAL
1	B	81	LYS
1	B	106	ASN
1	B	135	ASN
1	B	158	VAL
2	R	75	VAL
2	R	113	ILE
2	R	154	VAL

Continued on next page...

PRELIMINARY VALIDATION REPORT

Continued from previous page...

Mol	Chain	Res	Type
2	S	75	VAL
2	S	105	LYS
2	S	113	ILE
2	S	154	VAL

Sometimes sidechains can be flipped to improve hydrogen bonding and reduce clashes. All (6) such sidechains are listed below:

Mol	Chain	Res	Type
1	A	162	ASN
1	B	143	HIS
1	B	162	ASN
2	R	111	GLN
2	S	91	HIS
2	S	111	GLN

5.3.3 RNA (i)

There are no RNA molecules in this entry.

5.4 Non-standard residues in protein, DNA, RNA chains (i)

There are no non-standard protein/DNA/RNA residues in this entry.

5.5 Carbohydrates (i)

There are no monosaccharides in this entry.

5.6 Ligand geometry (i)

4 ligands are modelled in this entry.

In the following table, the Counts columns list the number of bonds (or angles) for which Mogul statistics could be retrieved, the number of bonds (or angles) that are observed in the model and the number of bonds (or angles) that are defined in the Chemical Component Dictionary. The Link column lists molecule types, if any, to which the group is linked. The Z score for a bond length (or angle) is the number of standard deviations the observed value is removed from the expected value. A bond length (or angle) with $|Z| > 2$ is considered an outlier worth inspection. RMSZ is the root-mean-square of all Z scores of the bond lengths (or angles).

PRELIMINARY VALIDATION REPORT

Mol	Type	Chain	Res	Link	Bond lengths			Bond angles		
					Counts	RMSZ	#|Z| > 2	Counts	RMSZ	#|Z| > 2
3	NAG	R	501	2	14,14,15	0.35	0	17,19,21	0.71	1 (5%)
3	NAG	B	178	1	14,14,15	0.37	0	17,19,21	0.63	0
3	NAG	A	178	1	14,14,15	0.33	0	17,19,21	0.65	1 (5%)
3	NAG	S	501	2	14,14,15	0.37	0	17,19,21	0.87	2 (11%)

In the following table, the Chirals column lists the number of chiral outliers, the number of chiral centers analysed, the number of these observed in the model and the number defined in the Chemical Component Dictionary. Similar counts are reported in the Torsion and Rings columns. '-' means no outliers of that kind were identified.

Mol	Type	Chain	Res	Link	Chirals	Torsions	Rings
3	NAG	R	501	2	-	0/6/23/26	0/1/1/1
3	NAG	B	178	1	-	0/6/23/26	0/1/1/1
3	NAG	A	178	1	-	0/6/23/26	0/1/1/1
3	NAG	S	501	2	-	0/6/23/26	0/1/1/1

There are no bond length outliers.

All (4) bond angle outliers are listed below:

Mol	Chain	Res	Type	Atoms	Z	Observed(°)	Ideal(°)
3	A	178	NAG	C1-O5-C5	2.33	115.35	112.19
3	R	501	NAG	C1-O5-C5	2.21	115.19	112.19
3	S	501	NAG	C1-O5-C5	2.21	115.18	112.19
3	S	501	NAG	O5-C1-C2	-2.05	108.06	111.29

There are no chirality outliers.

There are no torsion outliers.

There are no ring outliers.

No monomer is involved in short contacts.

5.7 Other polymers (i)

There are no such residues in this entry.

5.8 Polymer linkage issues (i)

There are no chain breaks in this entry.

PRELIMINARY VALIDATION REPORT

6 Fit of model and data (i)

6.1 Protein, DNA and RNA chains (i)

In the following table, the column labelled '#RSRZ> 2' contains the number (and percentage) of RSRZ outliers, followed by percent RSRZ outliers for the chain as percentile scores relative to all X-ray entries and entries of similar resolution. The OWAB column contains the minimum, median, 95th percentile and maximum values of the occupancy-weighted average B-factor per residue. The column labelled 'Q< 0.9' lists the number of (and percentage) of residues with an average occupancy less than 0.9.

Mol	Chain	Analysed	<RSRZ>	#RSRZ>2			OWAB(Å^2)	Q<0.9
1	A	122/128 (95%)	1.27	21 (17%)	1	1	56, 71, 96, 109	0
1	B	122/128 (95%)	1.13	11 (9%)	9	11	51, 73, 94, 116	0
2	R	96/147 (65%)	1.13	21 (21%)	0	0	70, 89, 132, 133	0
2	S	96/147 (65%)	1.16	14 (14%)	2	2	72, 90, 110, 115	0
All	All	436/550 (79%)	1.17	67 (15%)	2	2	51, 80, 111, 133	0

All (67) RSRZ outliers are listed below:

Mol	Chain	Res	Type	RSRZ
1	B	177	SER	11.6
1	B	176	ILE	7.5
1	A	176	ILE	7.2
1	A	175	PHE	4.4
2	S	113	ILE	4.1
2	R	113	ILE	3.7
2	R	110	PHE	3.5
2	R	73	MET	3.5
2	S	127	HIS	3.4
2	S	110	PHE	3.4
1	B	173	PRO	3.4
2	R	101	GLN	3.2
2	S	95	PRO	3.1
2	R	93	PRO	3.1
2	R	103	GLN	3.0
1	B	175	PHE	3.0
1	A	177	SER	3.0
2	S	140	LEU	2.9
2	R	77	PRO	2.9
2	R	92	HIS	2.8
1	B	144	VAL	2.8

Continued on next page...

PRELIMINARY VALIDATION REPORT

Continued from previous page...

Mol	Chain	Res	Type	RSRZ
2	R	137	PHE	2.8
2	R	75	VAL	2.8
1	B	73	SER	2.8
1	A	110	VAL	2.7
1	B	110	VAL	2.7
2	R	111	GLN	2.7
1	A	113	PHE	2.7
2	R	139	PHE	2.7
2	R	95	PRO	2.6
2	R	100	VAL	2.6
2	S	111	GLN	2.5
2	S	123	GLY	2.5
1	A	87	LEU	2.5
2	S	154	VAL	2.5
1	A	171	ALA	2.5
2	S	99	GLY	2.5
2	R	79	PHE	2.5
2	S	137	PHE	2.5
2	S	129	LYS	2.4
1	B	148	ILE	2.4
1	A	136	VAL	2.4
2	R	112	CYS	2.4
1	A	102	ALA	2.4
2	R	140	LEU	2.4
1	A	150	LEU	2.3
2	S	86	CYS	2.3
1	A	90	LEU	2.3
1	A	132	LYS	2.3
1	A	71	MET	2.3
2	S	90	ARG	2.3
2	S	126	GLY	2.2
2	R	156	GLY	2.2
1	A	79	VAL	2.2
1	A	169	LEU	2.2
2	R	129	LYS	2.2
1	B	89	ILE	2.1
1	A	82	VAL	2.1
2	R	96	PRO	2.1
1	A	124	ILE	2.1
1	A	167	ILE	2.1
2	R	127	HIS	2.1
1	A	101	VAL	2.0

Continued on next page...

PRELIMINARY VALIDATION REPORT

Continued from previous page...

Mol	Chain	Res	Type	RSRZ
1	A	158	VAL	2.0
1	B	95	TYR	2.0
1	B	174	GLN	2.0
1	A	78	CYS	2.0

6.2 Non-standard residues in protein, DNA, RNA chains (i)

There are no non-standard protein/DNA/RNA residues in this entry.

6.3 Carbohydrates (i)

There are no monosaccharides in this entry.

6.4 Ligands (i)

In the following table, the Atoms column lists the number of modelled atoms in the group and the number defined in the chemical component dictionary. The B-factors column lists the minimum, median, 95th percentile and maximum values of B factors of atoms in the group. The column labelled 'Q< 0.9' lists the number of atoms with occupancy less than 0.9.

Mol	Type	Chain	Res	Atoms	RSCC	RSR	B-factors(Å^2)	Q<0.9
3	NAG	B	178	14/?	0.76	0.14	109,111,111,111	0
3	NAG	S	501	14/?	0.76	0.21	93,94,95,95	0
3	NAG	R	501	14/?	0.78	0.23	97,99,99,100	0
3	NAG	A	178	14/?	0.86	0.21	106,109,109,109	0

6.5 Other polymers (i)

There are no such residues in this entry.

PRELIMINARY VALIDATION REPORT
